# Interventions in cytokine signaling: novel horizons for psoriasis treatment

**DOI:** 10.3389/fimmu.2025.1573905

**Published:** 2025-04-15

**Authors:** Lisha Li, Jun Liu, Jiaye Lu, Junchao Wu, Xinyue Zhang, Tianyou Ma, Xiying Wu, Quangang Zhu, Zhongjian Chen, Zongguang Tai

**Affiliations:** ^1^ Shanghai Skin Disease Hospital, School of Medicine, Tongji University, Shanghai, China; ^2^ Shanghai Engineering Research Center of Topical Chinese Medicine, Shanghai, China; ^3^ Beijing Key Laboratory of Molecular Pharmaceutics and New Drug Delivery Systems, State Key Laboratory of Natural and Biomimetic Drugs, School of Pharmaceutical Sciences, Peking University, Beijing, China

**Keywords:** psoriasis, inflammatory skin disease, immune cells, cytokines, cytokine network

## Abstract

Intricate interactions between immune cells and cytokines define psoriasis, a chronic inflammatory skin condition that is immunological-mediated. Cytokines, including interleukins (ILs), interferons (IFNs), tumor necrosis factors (TNFs), chemokines, and transforming growth factor-β (TGF-β), are essential for controlling cellular activity and immunological responses, maintaining homeostasis and contributing to the pathogenesis of psoriasis. These molecules modulate the immune microenvironment by either promoting or suppressing inflammation, which significantly impacts therapeutic outcomes. Recent research indicates that treatment strategies targeting cytokines and chemokines have significant potential, offering new approaches for regulating the immune system, inhibiting the progression of psoriasis, and reducing adverse effects of traditional therapies. This review consolidates current knowledge on cytokine and chemokine signaling pathways in psoriasis and examines their significance in treatment. Specific attention is given to cytokines like IL-17, IL-23, and TNF-α, underscoring the necessity for innovative therapies to modulate these pathways and address inflammatory processes. This review emphasizes the principal part of cytokines in the -pathological process of psoriasis and explores the challenges and opportunities they present for therapeutic intervention. Furthermore, we examine recent advancements in targeted therapies, with a particular focus on monoclonal antibodies, in ongoing research and clinical trials.

## 1 Introduction

Psoriasis, as a chronic and persistently relapsing skin disease, has not yet been cured (1). Characterized by its unique clinical manifestations, this disease is known for the appearance of scaly erythematous or patchy skin lesions on the skin, nails, and joints, which not only affect the patient’s appearance but can also be accompanied by itching and pain, severely affecting the quality of life (2). Based on clinical presentation, psoriasis is divided into four types: guttate psoriasis, pustular psoriasis, plaque psoriasis, erythrodermic psoriasis and psoriatic arthritis (PsA) (3–5). Each type exhibits specific pathological features and clinical manifestations, but they all share a common pathological basis of abnormal keratinocyte (KC) proliferation and a hyperactive immune system (6). While the exact cause of psoriasis remains uncertain, recent research emphasizes the pivotal role of cytokines in its development.

**Figure 1:**
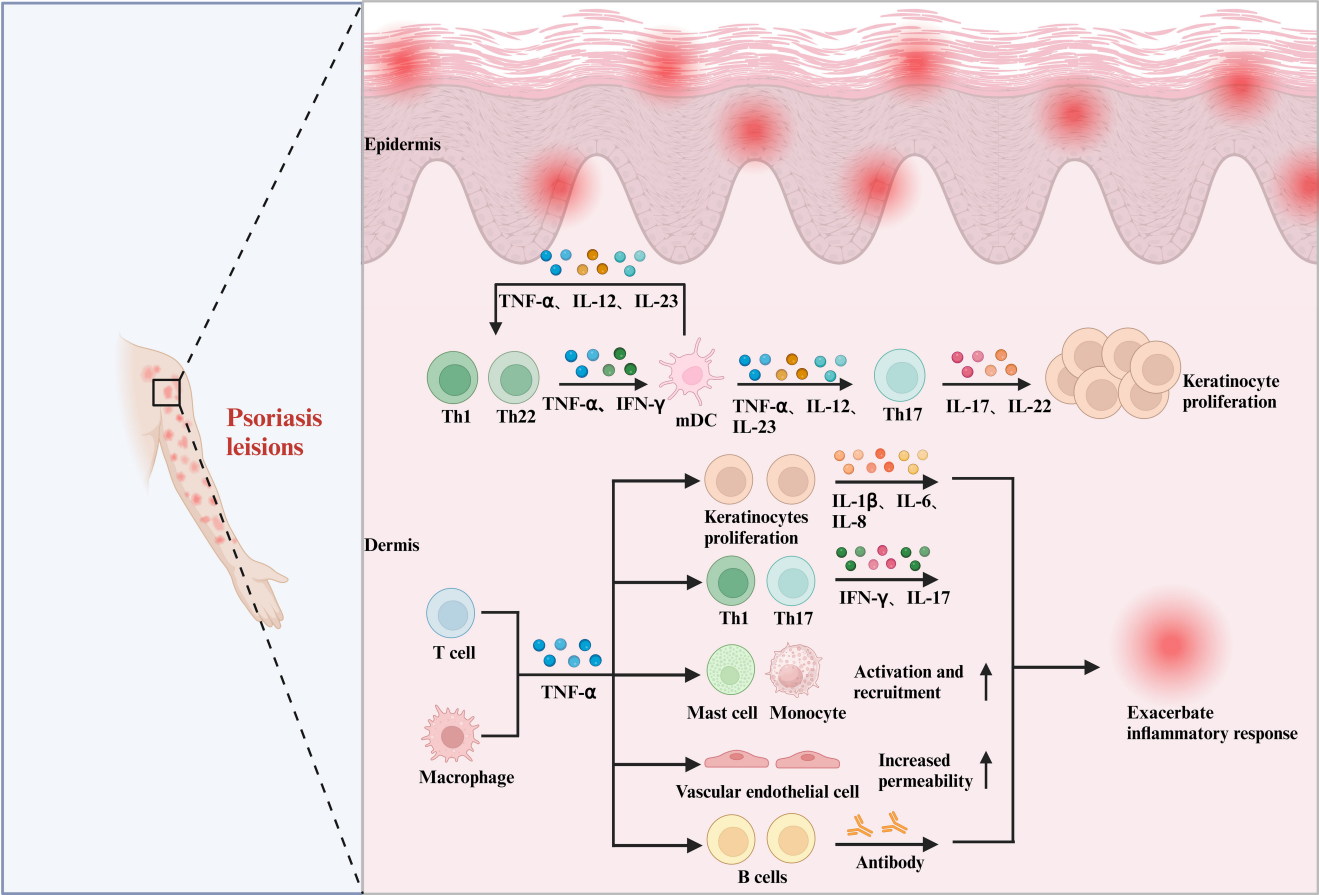
The pleiotropic effects of cytokines in psoriasis. In psoriasis, the role of TNF-α is equally significant and cannot be overlooked. Within psoriasis lesions, TNF-α secreted by T cells and macrophages interacts with DCs, keratinocytes, Th1 and Th17 cells, mast cells, monocytes, vascular endothelial cells, B cells, and others. This interaction promotes the downstream release of various inflammatory factors, including IL-17, IL-1β, and IL-6, which in turn leads to the abnormal proliferation of keratinocytes and exacerbates the inflammatory response.

Cytokines are diverse, low molecular weight proteins with pleiotropic biological activities, functioning in autocrine, paracrine, and endocrine manners. Produced by immune cells such as monocytes, macrophages, T cells, B cells, and natural killer cells, as well as non-immune cells like epithelial cells and fibroblasts, their production is triggered by external stimuli. By binding to their specific receptors, they regulate innate and adaptive immunity, hematopoiesis, cell growth, and tissue repair (7). Chemokines, a superfamily of small, secreted proteins within the cytokine family, are crucial for controlling inflammatory reactions because they attract immune cells to inflamed areas (8). In psoriasis, cytokines including tumor necrosis factor-alpha (TNF-α), interleukin-17 (IL-17), and IL-23, along with the chemokine CCL20, significantly contribute to disease initiation and progression (9–11). For instance, IL-17A can enhance the proliferative capacity of KCs, upregulate the expression of keratin 17 in KCs, while downregulating the production of filaggrin and diverse cell adhesion molecule-related genes, leading to structural damage of the epidermal barrier and triggering local psoriasis immune responses (12). Furthermore, IL-17A can stimulate KCs to express a variety of chemokines and upregulate the expression of IL-6, chemokine (C-X-C motif) ligand (CXCL) molecules in neutrophils, thereby chemoattracting a variety of immune cells to migrate to the psoriatic epidermal lesions (**Figure 1**) (13). It is evident that by inhibiting the activity of these molecules or blocking their signal transduction, inflammation can be effectively reduced, skin lesions decreased, and the quality of life for patients improved. The introduction of biologics, such as monoclonal antibodies targeting TNF-α, IL-17, and IL-23, has markedly improved psoriasis treatment by providing highly specific and effective treatment options. Nevertheless, some patients do not respond to these treatments, and prolonged use can lead to adverse effects, including a heightened risk of infections. Therefore, continued research and development of new targeted therapeutic strategies to improve efficacy, reduce side effects, and expand therapeutic options for individuals with psoriasis is a current focus of research.

This review offers a current and thorough investigation of cytokine and chemokine signaling pathways in psoriasis, highlighting their essential role in shaping therapeutic strategies amidst the rapidly changing landscape of psoriasis treatments. By discussing how these molecules influence immune responses in psoriasis, we have underscored the necessity of developing novel therapeutic agents capable of modulating these signaling pathways to effectively combat the inflammatory symptoms of psoriasis. The article also elaborates on the central role of cytokines in the pathological development of psoriasis, while analyzing the challenges and potential therapeutic opportunities they present. Furthermore, recent progress in targeted treatments is highlighted, particularly focusing on monoclonal antibodies currently under investigation in research and clinical trials, as well as promising targets in preclinical development. These studies significantly enhance our understanding of cytokines and chemokines in psoriasis treatment and support their potential for clinical application. The review provides a systematic summary and a comprehensive analysis, offering new insights and valuable references for the future development of specific therapeutic options for psoriasis.

## 2 Cytokine networks and their role in psoriasis

**Figure 2:**
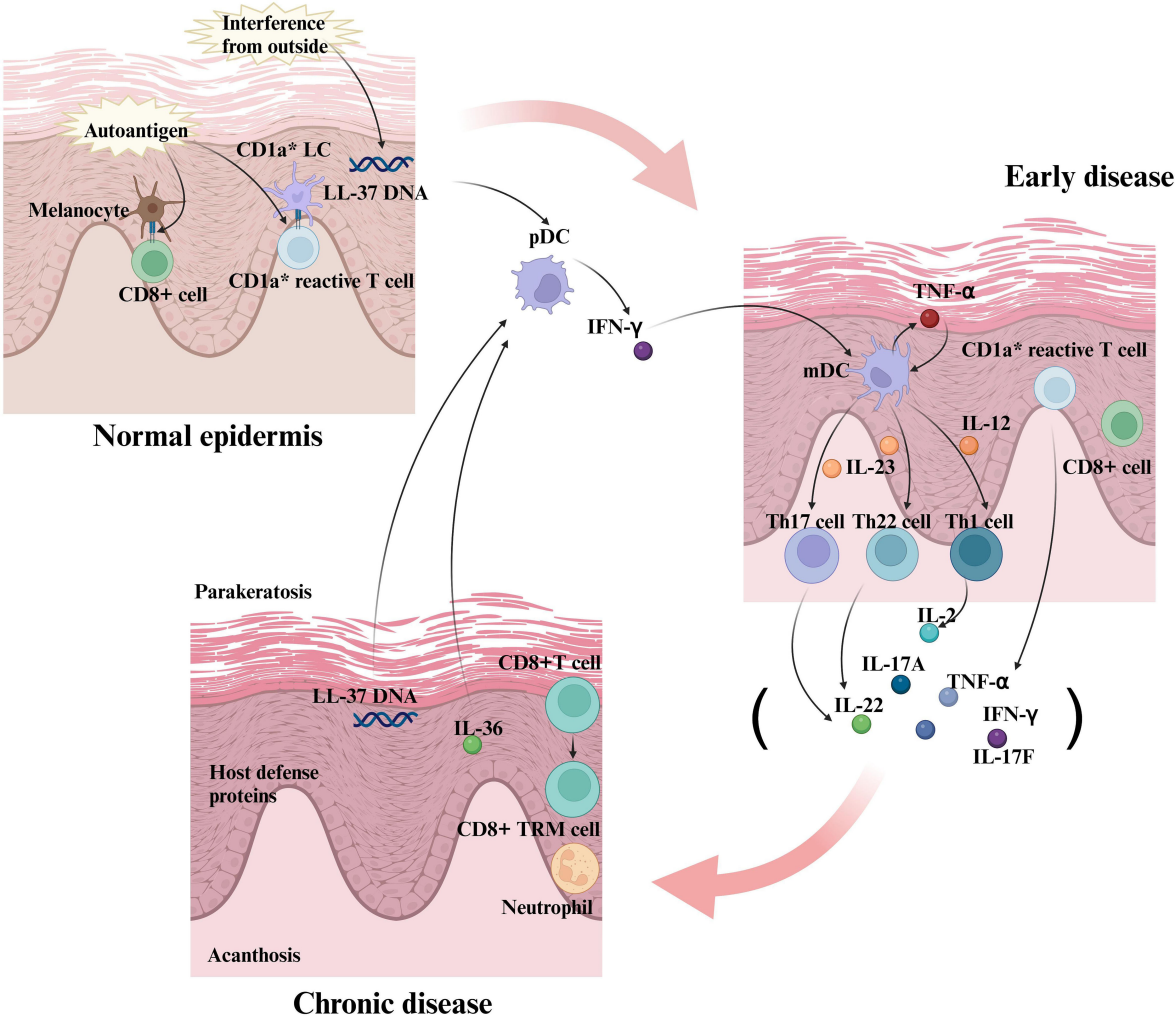
The formation of interactions between inflammatory factors and immune cells in the early stages of psoriasis and the creation of positive feedback loops. In the early stages of psoriasis, keratinocytes and immune cells (e.g., dendritic cells) release initial inflammatory factors. These inflammatory factors further activate and recruit immune cells, such as T cells, to the area of the skin lesion. In response to the inflammatory factors, the T cells are activated and differentiate into specific Th subpopulations, such as Th1, Th17, and Th22 cells. Th1 cells produce IL-2, Th17 cells produce IL-17, and Th22 cells produce IL-22, cytokines that further exacerbate the inflammatory response. Activated T cells interact with keratinocytes, endothelial cells, and dendritic cells to release more inflammatory factors, creating a positive feedback loop. Inflammatory factors such as TNF-α and IL-17 act directly on keratinocytes to promote their abnormal proliferation and differentiation, which are key features of lesion formation in psoriasis. In summary, the interactions between inflammatory factors and immune cells during the progression of psoriasis form a complex network that continually amplifies the inflammatory response through positive feedback mechanisms, leading to the development and persistence of skin lesions. Therapeutic strategies often target these key inflammatory factors and immune cells to reduce symptoms and control the disease.

The immune system functions as an intricate network, encompassing lymphoid organs, immune cells, humoral factors, and cytokines. Cytokines play a pivotal role within this network as small molecular weight proteins secreted by cells (14). These molecules can exist in both secreted and membrane-bound forms, and they are instrumental in regulating cell behavior and facilitating communication between cells. This communication is crucial for regulating diverse biological processes such as immune responses, inflammation, cell proliferation, and apoptosis (**Figure 2**) (15).

Cytokines can be classified into distinct categories based on their origin, structure, and function, each playing a distinct role in immune regulation. ILs, for example, are essential in modulating white blood cell activity and are involved in both immune defense and inflammatory processes (15). Similarly, interferons (IFNs) are generated in response to viral infections, exhibiting antiviral, antiproliferative, and immunomodulatory effects (16). TNFs play a role in regulating cell death, inflammation, and immune cell activation. Colony-stimulating factors (CSFs) encourage the growth and differentiation of blood cell precursors, whereas growth factors facilitate cell division and tissue repair. Chemokines are crucial for directing immune cells to sites of inflammation or infection, significantly contributing to the immune response (14). The interaction between cytokines and their receptors is also vital, as these receptors are proteins that bind to cytokines and initiate signal transduction pathways. In addition to these, there are specialized cytokines such as Thrombopoietin (TPO) and leukemia inhibitory factor (LIF), which have specific roles in the regulation of blood cell production and the inhibition of leukemia cells, respectively. This intricate interplay of components within the immune system ensures a robust defense against pathogens and maintains overall health (15).

Cytokines are indispensable in the regulation as well as function within immunopathology, and targeting various cytokines has become a potent approach in treating immune diseases, ranging from autoimmune to allergic conditions (17). The inflammatory process in psoriasis engages both the innate and adaptive immune mechanisms. The characteristic lesion of psoriasis is the hyperproliferation and dysregulated differentiation of epidermal KCs, a process that involves the action of numerous cytokine pathways, thereby triggering and maintaining the inflammatory pathways of psoriasis. Plaque psoriasis, the most prevalent form, is linked to TNF-α, IL-23, and IL-17 pathways, while pustular psoriasis, a less common type, is associated with IL-36RN genetic mutations. In chronic psoriasis, the adaptive immune system sustains the disease via the TNF-α/IL-23/IL-17 axis. The interaction between IL-23 and IL-17 is central to psoriasis pathogenesis, sustaining Th17 cell activity, which mainly produces IL-17. Cytokines, such as IL-23, IL-17, IL-22, and TNF-α, play a role in the pathogenesis of PsA (9, 18, 19). In psoriasis lesions, TNF-α exhibits pro-inflammatory properties that stimulate IL-12 and IL-18 production, influencing Th1 immune responses. IL-12 and IL-18 are potent inducers of IFN-γ, playing a crucial part in regulating Th1 immune responses. TNF-α is instrumental in the IL-23/IL-17 axis, working in synergy with IL-17A to regulate cytokines and keratinocyte-associated genes involved in psoriasis. Multiple studies have demonstrated that IFN-γ serves as a prognostic factor for psoriasis, with the IFN signaling pathway contributing to its pathogenesis (19).

The interplay between cytokines forms a complex network that finely regulates immune responses and inflammatory processes through positive and negative feedback mechanisms. The cytokine network intricately regulates immune cell recruitment, activation, and proliferation, alongside the abnormal proliferation and differentiation of KCs. These interactions are vital in psoriasis pathogenesis, where the DC-IL-23-Th17 cell-IL-17-KC axis forms a positive feedback loop that advances the disease. Specifically, mature DCs (mDCs) release IL-23, which boosts Th17 cell development and proliferation. In turn, IL-17 produced by Th17 cells further stimulates the production of IL-23, creating a self-amplifying inflammatory cycle (19). IL-10, an anti-inflammatory cytokine, inhibits IL-23 and IL-17 secretion, creating a negative feedback loop to suppress inflammation (18). The balance between pro-inflammatory and anti-inflammatory cytokines is indispensable for maintaining immune homeostasis and preventing the worsening of inflammatory diseases. A foundational component of immune control is the delicate balance within the cytokine network, and knowledge of these mechanisms can help create tailored treatments for immunological-mediated diseases.

## 3 Immunometabolic regulation and environmental triggers in psoriasis

Keratinocytes and immune cells (such as Th17 and macrophages) in psoriasis lesions exhibit significant lipid metabolism abnormalities.

### 3.1 Lipid metabolism reprogramming drives amplification of inflammatory signaling

#### 3.1.1 Cholesterol/fatty acid metabolism imbalance

Patients with psoriasis show elevated levels of oxidized low-density lipoprotein (oxLDL) in their serum. OxLDL activates the scavenger receptor CD36 on the surface of macrophages, triggering the assembly of the NLRP3 inflammasome and promoting the maturation and release of IL-1β (20) Additionally, the upregulation of fatty acid synthase (FASN) in keratinocytes leads to the accumulation of free fatty acids, which enhances IL-23 secretion via the TLR4/MyD88 pathway, thereby activating the IL-17 signaling axis (21).

#### 3.1.2 PPARγ signaling inhibition

The key lipid metabolism regulator PPARγ’s expression is reduced in psoriasis patients’ epidermis. This reduction leads to decreased synthesis of anti-inflammatory lipid mediators (such as 15d-PGJ2), which fails to inhibit NF-κB-driven transcription of TNF-α (22).

#### 3.1.3 SCD1 and lipotoxic inflammation

The expression of stearoyl-CoA desaturase 1 (SCD1) is significantly upregulated in psoriasis lesions. SCD1 catalyzes the conversion of saturated fatty acids (such as palmitic acid) to monounsaturated fatty acids (such as oleic acid). Accumulation of palmitic acid activates the endoplasmic reticulum stress (ERS) signaling pathway (PERK/eIF2α) in keratinocytes, inducing the expression of IL-23 and IL-17F and exacerbating epidermal hyperproliferation (23).

#### 3.1.4 SREBP Pathway dysregulation

Sterol regulatory element-binding protein (SREBP1c) is abnormally activated in psoriasis, promoting the transcription of fatty acid synthesis-related genes (such as ACC and FASN), leading to excessive lipid droplet deposition. Lipid droplets release arachidonic acid derivatives (such as prostaglandin E2, PGE2), which activate the cAMP-PKA pathway dependent on the EP4 receptor, inhibiting Treg function and promoting Th17 differentiation (24).

### 3.2 Oxidative stress and inflammatory signaling in a vicious cycle

In psoriatic epidermis, the inhibition of the Nrf2-Keap1 pathway results in reduced nuclear translocation of Nrf2 and downregulation of antioxidant response element (ARE)-driven genes, such as HO-1 and NQO1 (25), leading to decreased reactive oxygen species (ROS) clearance capacity and further amplification of NF-κB and STAT3 signaling. Additionally, mitochondrial ROS cause oxidative damage to mitochondrial DNA (mtDNA), triggering its release into the cytoplasm. This mtDNA activates the cGAS-STING pathway, inducing the production of type I interferons (IFN-α/β) (26), which synergize with IL-17A to promote abnormal keratinocyte proliferation. Furthermore, ROS-induced inhibition of DNA methyltransferase (DNMT) activity leads to hypomethylation of the SOCS3 gene promoter, impairing its negative regulation of the JAK/STAT pathway and exacerbating IL-6/IL-23 signaling (26).

### 3.3 Environmental triggers and cytokine network interactions

#### 3.3.1 Dysbiosis of the microbiome reconfigures immune-epidermal dialogue

In psoriasis, disruption of the gut-skin axis is characterized by an increased abundance of Prevotella in the gut microbiota and a corresponding reduction in short-chain fatty acids (SCFAs), leading to compromised intestinal barrier integrity. This facilitates the translocation of endotoxins into the bloodstream, activating monocytes via TLR2/4 and promoting IL-23 secretion (27). Meanwhile, the overproliferation of Malassezia spp. can degrade sebum to produce free fatty acids, such as oleic acid, disrupting the epidermal barrier and activating the CARD14 signaling pathway in keratinocytes. This leads to the release of IL-36γ, which recruits IL-17A^+^ γδ T cells (28). Besides, reduced colonization of Staphylococcus epidermidis weakens its ability to induce Tregs to produce IL-10, indirectly enhancing the pathogenic effects of Th17 cells via IL-17A (29).

#### 3.3.2 Chronic stress exacerbates inflammation via the neuro-immune axis

Chronic stress contributes to the pathogenesis of psoriasis through several neuroimmune pathways. Activation of the sympathetic nerve system stimulates dermal DCs via the β2-adrenergic receptor (β2-AR), promoting IL-23 secretion while inhibiting IL-10 expression in Tregs, thereby creating a Th17-polarized microenvironment (30). Chronic psychological stress dysregulates the hypothalamic-pituitary-adrenal (HPA) axis, reducing the sensitivity of glucocorticoid receptors (GR) and impeding cortisol’s ability to effectively inhibit the production of TNF-α and IL-1β in macrophages (31). In addition, stress-induced overexpression of nerve growth factor (NGF) enhances the excitability of sensory neurons via the TrkA receptor, promoting the release of calcitonin gene-related peptide (CGRP). This directly stimulates mast cell degranulation, releasing TNF-α and IL-33, and amplifying the IL-17/IL-23 axis (31).

## 4 Cytokines in different types of psoriasis

As mentioned earlier, psoriasis can be divided into these subtypes: guttate psoriasis, pustular psoriasis, plaque psoriasis, erythrodermic psoriasis and psoriatic arthritis (PsA). There are specific differences in the cytokine-driven mechanisms of different psoriasis subtypes: each pathologic subtype corresponds to a characteristic cytokine profile. Their mediated immune signaling networks and tissue damage patterns show significant heterogeneity. This section focuses on the different psoriasis subtypes and their relationship to cytokines.

### 4.1 Guttate psoriasis

Guttate psoriasis presents as tear-drop erythematous scaly lesions scattered throughout the body and is most common in children and adolescents. Treatment is centered on long-term remission and lesion clearance is achieved through a stepwise regimen. This includes a combination of topical agents (glucocorticoids/retinoids) and oral immunomodulators; narrow-spectrum UVB or PUVA photochemotherapy to modulate aberrant epidermal proliferation; and novel biologics targeting key pathways such as TNF-α/IL-17/IL-23. The clinical pathway emphasizes the selection of individualized regimens based on age, disease duration and extent of skin lesions, combined with multimodal therapy to maintain long-term remission status (32). The disease exhibits genetic susceptibility, and enhanced polymorphisms in genes such as human leukocyte antigen (HLA)-Cw6 may be associated with an increased risk of immune abnormalities. HLA-Cw6 positivity has been linked to guttate psoriasis, while HLA-Cw1 positivity has been associated with erythrodermic psoriasis, pustular psoriasis, and axial psoriatic arthritis (33, 34). Notably, plaque psoriasis and guttate psoriasis exhibit significant differences in T cell phenotype and function. In the skin lesions and peripheral blood of patients with plaque psoriasis, the number of Foxp3^+^ Tregs is higher than in patients with guttate psoriasis. Conversely, patients with guttate psoriasis have a higher proportion of IL-17^+^ CD4^+^ cells compared to those with plaque psoriasis (35). Furthermore, IFN-γ and IL-17 derived from CD4^+^ T cells play a pivotal role in the pathogenesis of guttate psoriasis (36, 37). Interestingly, in HLA-Cw6^+^ patients experiencing guttate psoriasis flare-ups associated with pharyngitis, the Th17-associated response is more pronounced, leading to significantly elevated levels of IL-17A, IL-17F, and IL-6, which are involved in Th17 differentiation (38). Additional studies have indicated that IL-9 also plays a crucial role in guttate psoriasis by upregulating IL-17A through its impact on cutaneous lymphocyte-associated antigen (CLA)^+^ T cell viability, thereby promoting inflammatory responses in guttate psoriasis (34, 39).

### 4.2 Pustular psoriasis

Pustular psoriasis is a rare subtype of psoriasis marked by sterile neutrophilic pustules, which can be fatal in some cases (40). Traditional therapies include oral retinoids, cyclosporine, and methotrexate, however, all are based on plaque psoriasis experience (41). There are no GPP-specific drugs in the world (especially in Europe and the United States), and some countries have the conditions to use the existing therapies. Anti-TNF/IL-17A drugs (e.g., infliximab, sukinumab) are effective in only some patients, and IL-17 inhibitors combine ease of injection with low side effects (42). IL-36 (IL-1 family member) acts on keratinocytes, immune cells, etc. via an autocrine/paracrine mechanism, and its receptor complex (IL-36R/IL-1RAcP) triggers inflammatory signaling. Imbalance between IL-36 proinflammatory isoforms (α/β/γ) and the antagonist IL-36Ra drives an aberrant inflammatory cascade, inducing the release of proinflammatory factors, neutrophil chemotactic factors and antimicrobial peptide release. The simultaneous activation of dendritic cells and T cells creates a vicious cycle. Dysregulation of this pathway is a central mechanism of GPP pustule formation and tissue damage, and targeting IL-36R blockade is a potential therapeutic direction (42, 43). The IL-1β inhibitor gevokizumab demonstrated preliminary efficacy in 2 cases, suggesting its potential therapeutic value. Focusing on the IL-36/IL-1 pathway, we are promoting the clinical translation of precision biologics to break through the bottleneck of refractory GPP treatment (44).

### 4.3 Plaque psoriasis

Plaque psoriasis is the most common subtype of psoriasis (11). It is characterized by well-demarcated papular scaly lesions, which appear as round or oval infiltrated plaques covered with silvery-white mica-like scales. It is often accompanied by significant itching, which in severe cases may lead to epidermal cracking and spot bleeding (45). TNF-α, IL-17 and IL-23 constitute the core inflammatory axis in psoriasis. Genetic/environmental factors activate plasmacytoid dendritic cells (pDC), which release TNF-α, IFN-α/γ, and activate mDC, which produce large amounts of IL-12/IL-23 in psoriatic lesions, and accordingly drive the differentiation of Th1/Th17 cells. Th1 cells release TNF-α, and Th17 is the main source of IL-17 that directly stimulates the abnormal proliferation of KC. Activated KCs produce more IL-1β, TNF-α and chemokines, recruiting immune cells to form a self-sustained inflammatory network (18, 46, 47). In addition, it has been claimed that pathogenic memory T cells retain the ability to generate IL-17A for a long period, exacerbating the chronic course of the disease (48). Traditional targets have focused on TNF-α, IL-12, and IL-17/receptor inhibitors. More recently, biologics of IL-23 have emerged (49). There is evidence that drugs targeting the p19 subunit of IL-23 (e.g. Risankizumab) significantly enhance efficacy durability, with excellent 4-year drug survival data (50).

### 4.4 Erythrodermic psoriasis

Erythrodermic psoriasis is a rare and severe subtype of psoriasis that presents with generalized cutaneous inflammation and immune disorders with high mortality (51). Existing conventional treatments include glucocorticoids and cyclosporine, which are effective in the short term but are prone to relapse and have limited efficacy. Erythrodermic psoriasis shares the TNF/IL-17A inflammatory axis with plaque psoriasis, but there is a unique immunophenotype of Th2/Th17 coactivation in erythrodermic psoriasis. Th17 cell infiltration is predominant in the lesion area and IL-17A is the common core pathway (shared with plaque type). Some studies have shown significant elevation of serum IgE, IL-4, and IL-13, suggesting the involvement of Th2 immune abnormalities in the pathologic process. Biologic anti-TNF (e.g., Etanercept), anti-IL-17A (e.g., Skuticilumab), and anti-IL-12/23 agents (e.g., Ustekinumab) have demonstrated therapeutic potential in studies (52). In addition, Deucravacitinib is the world’s first oral highly selective TYK2 inhibitor, which provides a novel targeted treatment option for severe erythrodermic psoriasis by precisely inhibiting pathogenic cytokine signaling such as IL-12/IL-23 through targeting and regulating TYK2 kinase in the JAK-STAT signaling pathway (53).

### 4.5 Psoriatic arthritis

PsA is a chronic immune joint inflammatory disease involving the joints, skin, nails and spine and is a common comorbidity of psoriasis. PsA is associated with elevated cardiovascular risk. Approximately 30% of patients with psoriasis progress to PsA (54), and 40% have an inadequate response to conventional therapy (55). Joint inflammation is dominated by the IL-23/IL-17 axis, which is activated by IL-23 to activate Th17 cells that secrete IL-17/IL-22/TNF-α, triggering inflammation, bone destruction, and tissue damage (56). Skin/joint innate immune cells trigger the IL-12/IL-23 pathway, expanding Th1/Th17 cells and perpetuating the inflammatory cycle (55). IL-23/IL-17 pathway-targeted agents (e.g., Ustekinumab, Stavudineumab) are effective in psoriasis, but have relatively limited improvement in PsA (55, 57).

## 5 The interleukin family

### 5.1 IL-23

Monoclonal antibodies that specifically suppress IL-23 have been demonstrated to significantly reduce or even completely eradicate skin lesions in psoriasis patients, suggesting that IL-23 are fundamental to the development of psoriatic lesions (58). IL-23, a heterodimeric protein composed of p19 and p40 subunits linked by disulfide bonds, shares structural similarities with IL-12, leading to analogous roles in T-cell-mediated immune responses (59–61). Psoriatic lesions reveal significantly higher levels of IL-23 expression than normal skin, notably in the affected areas’ epidermis and dermis (62). IL-23 is primarily derived from immune cells within the dermis of psoriatic lesions, notably macrophages and bone mDCs, as well as other cellular subsets (63, 64). Thus, further exploration is necessary to elucidate how the function of these cells can be modulated to decrease IL-23 secretion and to discern the consequent effects on the disease evolution of psoriasis.

Th17 cells, a type of T lymphocyte pivotal for secreting inflammatory cytokines like IL-17, IL-22, IL-26, and TNF-α, are primarily activated by IL-23 to trigger inflammatory and immune responses. In patients with psoriasis, a significant elevation in the number of Th17 cells is observed within the skin tissue, and the inflammatory cytokines they secrete can induce the growth and proliferation of KCs, leading to the hyperplasia of the skin’s keratin layer and the formation of scaly lesions (65, 66). Under the influence of IL-23, Th17 cells generate an auto-amplifying feedforward inflammatory response in KCs and increase their number and vitality, accelerating the progression of skin lesions (63, 67–69). IL-23 promotes the infiltration and activation of inflammatory cells, worsening psoriasis, in addition to affecting Th17 cells. IL-23 promotes the infiltration and activation of various inflammatory cells, such as neutrophils, monocytes, macrophages, and DCs. Activated inflammatory cells secrete cytokines such as TNF-α, IL-1β, and IL-6, which enhance inflammatory and immune responses in the skin, leading to tissue damage and pathological changes (69–71).

IL-23 mediates the dysfunction of endothelial cells and KCs in psoriasis patients, accelerating KC proliferation and thereby contributing to disease progression. Psoriatic lesions are characterized by high microvascular permeability and angiogenesis, which are further promoted by IL-23 through the stimulation of endothelial cell proliferation and secretion, as well as neovascularization and vasodilation within the skin tissue, ultimately exacerbating the cutaneous inflammatory response and pathological changes (72). The application of selective VEGF inhibitors has notably improved skin lesions in mice with psoriasis (73). In contrast, introducing VEGF transgenically into mouse skin results in severe inflammatory dermatosis, resembling human psoriasis (74, 75). IL-23 also influences the cytoskeletal structure and intercellular junctions of KCs, inducing hyperproliferation and aberrant differentiation. These cellular alterations lead to stratum corneum thickening and keratinocyte accumulation, forming psoriasis’s characteristic lesions (9, 68, 76). Consequently, research targeting IL-23 has become a focal point in the current drug development strategy for psoriasis. Selective IL-23 inhibitors are increasingly used to treat psoriasis and other immune-mediated diseases. These therapeutics specifically target and inhibit IL-23 activity, thereby reducing Th17 cell differentiation and proliferation, and ultimately ameliorating psoriasis (76, 77). Furthermore, some novel IL-23 inhibitors are currently being investigated and hold promise for more effective outcomes in psoriasis treatment.

### 5.2 IL-17

Six members of the IL-17 family have been identified thus far, including IL-17A, IL-17B, IL-17C, IL-17D, IL-17E, and IL-17F. Notably, IL-17A shares approximately 55% homology with IL-17F, is usually co-expressed, and possesses similar biological functions (78). IL-17A was traditionally thought to be primarily secreted by Th17 cells. Recent evidence indicates that various cells, such as mast cells, γδ T cells, αβ T cells, and innate lymphoid cells found in psoriasis skin lesions and synovial fluid, can also produce IL-17A (79, 80). IL-17RA acts as a shared receptor for IL-17A, IL-17F, IL-17C, and IL-17E, partnering with another receptor subunit (IL-17RB, IL-17RC, IL-17RD, or IL-17RE) to enable specific signal transduction and initiate diverse inflammatory responses. These reactions involve the recruitment of inflammatory cells and the production of pro-inflammatory cytokines, such as IL-6, IL-8, and TNF-α, which contribute to the development and progression of various inflammatory conditions (81, 82).

**Figure 3:**
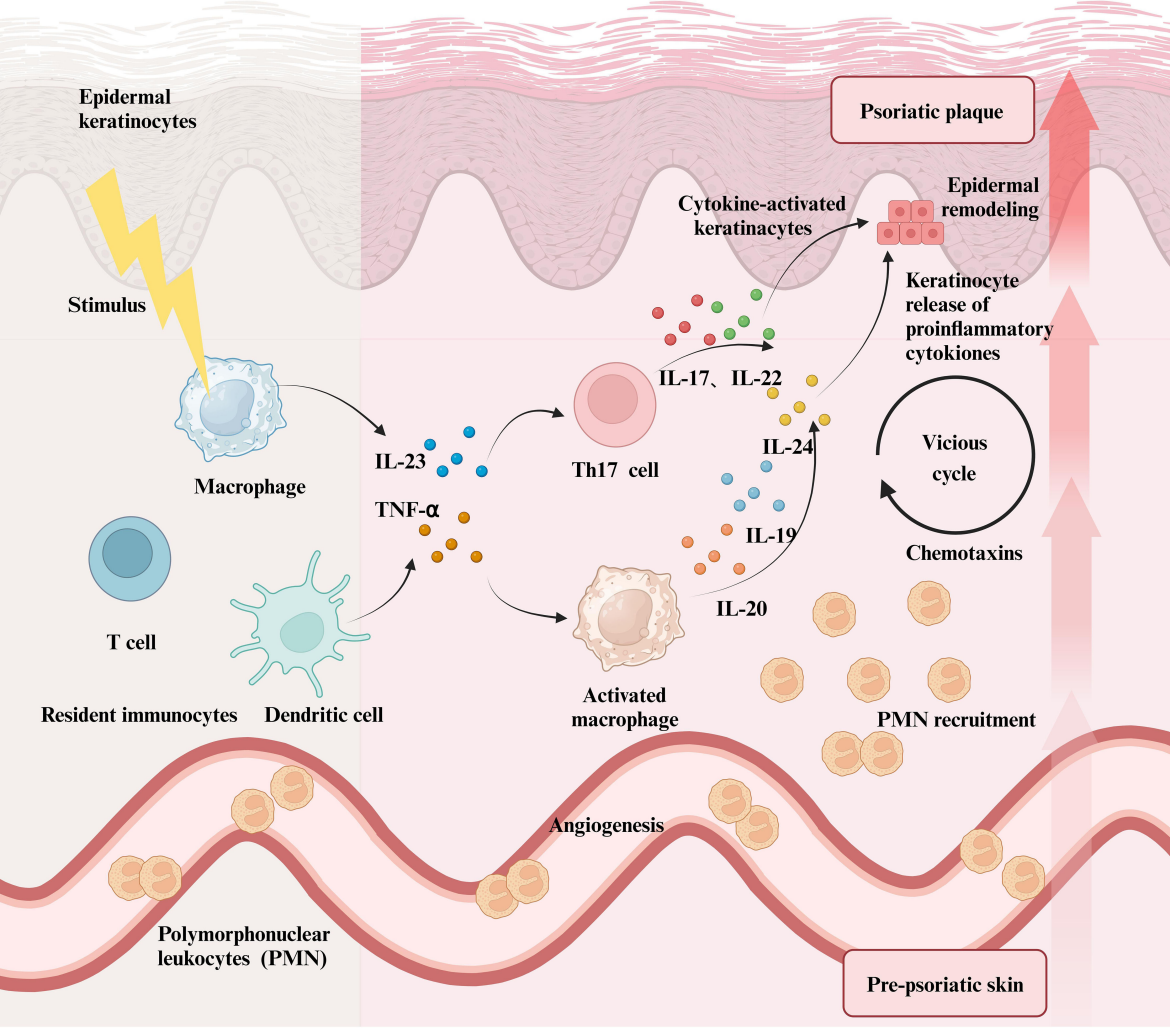
The role of immune cells in Psoriasis and the resulting inflammatory Cascade. When immune cells, including macrophages, DCs, and T cells, in healthy skin are stimulated by exogenous physicochemical factors, they secrete a significant number of inflammatory mediators such as TNF-α and IL-23. This not only activates macrophages, prompting them to release large quantities of inflammatory interleukins but also promotes the differentiation of T cells into Th17 cells, which in turn secrete IL-17, IL-22, and other cytokines. All of these inflammatory factors stimulate keratinocytes, leading to their abnormal proliferation and an inflammatory response in the skin. In psoriasis lesions, polymorphonuclear leukocytes further amplify local inflammatory reactions by releasing various inflammatory chemokines. Ultimately, the symptoms of psoriasis and the level of inflammation intensify, resulting in more severe lesions driven by multiple factors.

Numerous studies conducted over the last ten years have highlighted the significant function of IL-17A in regulating the adaptive as well as innate immune responses, distinguishing it as an imperative cytokine associated with the etiology of psoriasis and PsA (83, 84). Numerous cell types, such as fibroblasts, endothelial cells, and KCs, are triggered by IL-17A. In psoriasis patients, IL-17A levels are notably increased at skin lesions and nearby tissues, correlating positively with lesion severity (85) and KCs are the primary targets of IL-17A (86). Within KCs, IL-17A stimulates the production of various chemotactic factors, including CXCL1 and CXCL8, which recruit neutrophils, and CXCL20, which further attracts Th17 cells, Th22 cells, and mDCs. This process modulates immune cell function, contributing to the incidence and deterioration of psoriasis symptoms (87, 88). IL-17A not only enhances KC proliferation and keratinization but also stimulates immune cells to release inflammatory mediators such as TNF-α, IL-6, and IL-8, thereby amplifying inflammatory responses (84, 86, 89). Moreover, IL-17A principally drives the expression of four key cytokines: IL-36, IL-17C, IL-20, and IL-19, each possessing distinct functions in psoriasis (**Figure 3**). For instance, IL-36 and IL-17C mainly act synergistically to enhance tissue inflammation (90–92); overexpression of IL-20 can lead to epidermal changes characteristic of psoriasis (93–95); and IL-19 potentiates the effects of IL-17A via a positive feedback mechanism (96, 97). IL-17A enhances cutaneous inflammatory responses by activating several inflammation-related signaling pathways, such as STAT, NF-κB, and MAPK (98–101). The impact of IL-17A on psoriasis is modest by itself, but its significant influence in the disease’s development and progression is enhanced through synergy with TNF-α, IL-22, and IFN-γ (102). In psoriasis, peripheral neutrophils are elevated, recruited by IL-17E and CXCL8, and release IL-17A and extracellular vesicles, stimulating KCs to produce pro-inflammatory factors, enhance migration, and intensify inflammation. Infiltrating neutrophils highly express MMP-9, increasing vascular permeability and facilitating CD4^+^ T cell infiltration. While neutrophils do not directly express IL-17A, they can accumulate and release it via extracellular traps (103). The IL-23/IL-17 axis activates NF-κB, promotes the production of G-CSF, GM-CSF and chemokines (CXCL1, CXCL2, CXCL5, CXCL8), and facilitates neutrophil recruitment and mobilization (104). Neutrophil-derived IL-17 may be a target for IL-17A inhibitors that block its crosstalk with KCs and reduce inflammatory infiltration (79, 105). CXCL12^+^ Fibroblasts are located in the reticular dermis, express adipocyte markers, and respond to IL-17 and TNFα. The release of CXCR2 ligand and CXCL12 via the NFKBIZ pathway promotes neutrophil infiltration. Similar cells are highly expressive of neutrophil chemokines in human psoriatic skin and decrease after IL-17-targeted therapy (106).

Targeting the pivotal role of IL-17 in psoriasis, a variety of drugs that focus on IL-17 are currently under intensive basic and clinical research. Monoclonal antibodies against IL-17A and IL-17RA have been developed and proven effective in alleviating psoriasis symptoms. Furthermore, small molecule inhibitors aimed at the IL-17 signaling pathway are currently being investigated, presenting potential novel therapeutic options for the disease (1, 9, 107). In conclusion, more investigation needs to be conducted to clarify the mechanisms in which IL-17A causes psoriasis, which will contribute to the creation of innovative remedies.

The roles of other IL-17 family members should not be neglected alongside IL-17A. Research suggests IL-17C could be an effective biomarker for systemic anti-psoriasis therapy in moderate to severe psoriasis patients (108). IL-17F is known to contribute to the onset of psoriasis, while other IL-17 family members show a weaker link to disease progression, necessitating further exploration (109). Understanding the various functions of the IL-17 cytokine family is pivotal for clarifying psoriasis pathogenesis and developing new drugs.

### 5.3 The interleukin-1 superfamily

#### 5.3.1 IL-6

IL-6 plays a crucial part in inflammatory responses, with its biological activity being realized through binding to IL-6R and gp130 (110). IL-6 binding to IL-6R triggers gp130 homodimerization, resulting in the formation of an IL-6/IL-6R/gp130 complex. The complex then activates the Janus kinase (JAK) pathway, which subsequently triggers the STAT3, MAPK, and NF-κB pathways (111, 112). IL-6, a key inflammatory marker, is predominantly released by Th2 and antigen-presenting cells during immunological inflammation (113, 114). RORγt, a transcription factor specific to Th17 cells, plays a vital role in their differentiation and stabilization and IL-6 activates the STAT3 signaling pathway to enhance RORγt expression (115). Fibroblasts in psoriatic lesions release more IL-6 and mediate epidermal hyperplasia in conjunction with cytokines and growth factors such as IL-4 and IL-8 (116). Additionally, through suppressing the differentiation of RORγt in other T cell subsets, like Th 1 and Treg cells, IL-6 indirectly promotes the formation of Th17 cells and facilitates to preserve the relative preponderance of Th17 cells. The interaction between IL-6 and IL-17 in an inflammatory environment creates a positive feedback loop, enhancing the inflammatory response.IL-6 promotes IL-17A secretion, which subsequently triggers the release of inflammatory cytokines like IL-1β and TNF-α, intensifying inflammatory responses (117). Research suggests that IL-1β and TNF-α can promote Th17 cell differentiation facilitated by TGF-β and IL-6 (118). IL-6 enhances IL-23’s effect on Th17 cells, boosting IL-17 production and sustaining Th17 cell activity (119, 120). IL-6 is closely linked with IL-17A/Th17 in immune-mediated inflammation, affecting Th17 cell differentiation and inflammatory response progression through various pathways. This furthers our comprehension of its role in autoimmune disorders like psoriasis.

#### 5.3.2 IL-36

IL-36, a member of the IL-1 family, is expressed and functions in an autocrine or paracrine manner on various cells. Their influence encompasses KCs (121), epithelial cells, and immune cells (122). The IL-36 pathway, which includes IL-36α, IL-36β, IL-36γ, and their antagonists IL-36Ra and IL-38, serves as a crucial regulatory mechanism (123). Under certain conditions, aberrant activation or signaling of IL-36 can result in exorbitant inflammatory responses and neutrophil aggregation (124). Dysregulated expression of components within the IL-36 pathway can potentially initiate a positive feedback loop. For example, the excessive production of IL-36 pathway components may stimulate immune cells to secrete inflammatory cytokines, thereby amplifying the inflammatory response (125–128). This can stimulate the expression of chemokines like CCL13, CCL19, CXCL1, and CXCL12, which direct immune cells, especially neutrophils, to the site of inflammation. Neutrophil influx and chemokine release can damage skin tissue, promoting psoriasis progression. The presence of this positive feedback loop can disrupt the balance of the immune response, thereby impacting skin health. GPP is mainly marked by the abnormal activation of IL-36 (129). Mutations in several genetic loci are associated with GPP, such as Caspase Recruitment Domain 14 (CARD14), Adaptor Protein 1 Complex Sigma 3 Subunit (AP1S3), TNFAIP3 Interacting Protein 1 (TNIP1), and Serpin Family A Member 3 (SERPINA3) (130, 131). These genes are involved in the IL-1 and IL-36 signaling pathways, highlighting the importance of IL-36 in GPP. The IL17D-DDX5-IL-36R regulatory mechanism includes IL-36 and involves IL-17D, a member of the IL-17 family, with DDX5 being a protein-coding gene from the DEAD-box protein family (132, 133) and IL-36R mediates signaling for IL-36α, IL-36β, and IL-36γ (134). In psoriatic lesions, the expression of IL-17D is markedly elevated, promoting IL-36R-mediated signaling by suppressing the expression of DDX5 and further selectively amplifying the inflammatory response mediated by IL-36 (132). In addition, tissue protease S expressed by SFRP2^+^ fibroblasts activates IL-36G in keratinocytes, further exacerbating inflammation (122). In summary, IL-36 cytokines exert a crucial function in skin immunity, particularly in maintaining the skin barrier and modulating inflammation.

#### 5.3.3 IL-38

IL-38, a member of the IL-1 family, demonstrates anti-inflammatory effects by suppressing pro-inflammatory cytokine secretion (135). It regulates IL-36 via direct and indirect pathways. Direct actions involve suppressing IL-8 expression induced by IL-36 in PBMCs and reducing the pro-inflammatory effects of IL-36γ on KCs (136). Indirectly, IL-38 reduces IL-36γ release by modulating TLR signaling. Secondly, the downregulation of IL-17 and related mediators associated with Th38 differentiation increases the rebound effect of IL-36, meaning that the differentiation of Th38 may reduce the release of IL-36 (137). The X-linked receptor accessory protein-like 1 gene on the X chromosome is believed to mediate IL-38’s inhibitory effects on γδ T cells. Activation of γδT cells upregulates this gene, boosting IL-17 and IL-38 synthesis and indirectly reducing IL-36 release (138).

### 5.4 Other interleukin members

#### 5.4.1 IL-9

IL-9, a cytokine synthesized by diverse immune cells, like T lymphocytes, B lymphocytes, mast cells, and macrophages, exhibits elevated expression levels in the skin tissue of psoriasis patients (139). This rise suggests a possible link between IL-9 and disease pathogenesis (140, 141). IL-9 is mainly produced by Th9 cells, Th17 cells, Treg, and type 2 innate lymphoid cells (ILC2). IL-9 possesses an essential task in controlling immunological responses, especially when it concerns T cell-mediated immunity (142). Th9 cells are the primary producers of IL-9, independently contributing to allergic inflammation. Th9 cell differentiation is mainly regulated by IL-4/STAT6, IL-2/STAT5, and TGF-β signaling pathways (143). In the advancement of psoriasis, the excessive activation and differentiation of Th17 cells are critical contributors. Through direct or indirect procedures, IL-9 may alter the emergence and activity of Th17 cells, consequently influencing the course of skin inflammation. A potential cause is that IL-9 binds to a receptor complex made up of IL-9RA and the common gamma chain, which engages the MAPK, phosphoinositide 3-kinase (PI3K), and Jak-STAT signaling networks (144). An increased inflammatory response may arise from the transformation and growth of Th17 cells being aided by the activation of multiple pathways of signaling. Additionally, IL-9 may indirectly influence Th17 cell activation by modulating the production of cytokines or signaling molecules from other immune cells. For instance, IL-9 stimulates neighboring cells to produce pro-inflammatory factors like IL-23, thereby augmenting the development and functionality of Th17 cells (145). Excessive production of IL-9 may be linked to the hyperactivation of immune cells and dysregulated inflammatory responses, therefore contributing to the etiology of inflammatory skin conditions, especially psoriasis.

#### 5.4.2 IL-12

Macrophages, DCs, and fibroblasts produce the cytokines IL-12 and IL-23, both part of the IL-12 family (146). Their synthesis is regulated by multiple factors, such as gene expression variations, patterns of TLR expression, and the cross-regulation between different immune cell populations. This process also involves the secretion of other cytokines, like IL-10 and IFN-γ. IL-12, composed of IL-12α and IL-12β subunits, functions by interacting with IL-12Rβ1 and IL-12Rβ2 receptors. This interaction activates JAK2 and TYK2, resulting in the phosphorylation of STAT4 and other STAT family members, ultimately inducing IFN-γ secretion (146). IL-23, consisting of the IL-12β and IL-23α subunits, is elevated in psoriatic lesions compared to healthy skin (147). Although IL-12 and IL-23 are structurally similar, they have different functions. IL-23 interacts with the IL-12Rβ1 and IL-23R complex, primarily activating STAT3. This activation enhances the production of IL-17A, IL-17F, and IL-22, and supports the stabilization of Th17 cells (148, 149).

#### 5.4.3 IL-22

Several immune cell sorts, involving Th17 cells, γδT cells, natural killer T cells (NKT cells), and macrophages, manufacture IL-22, with Th17 cells being the principal source (150). In the dermis of both humans and mice, γδ T cells expressing the T cell marker CD69 have been observed, contributing to the promotion of Th17 cell differentiation (151). Th17 cells produce various cytokines, including IL-17A, IL-17F, and IL-22 (152). IL-22R, the receptor for IL-22, is predominantly expressed on epithelial cells and primarily facilitates the IL-22 signaling cascade (153). Under physiological conditions, IL-22 is essential for preserving the integrity of the epidermal barrier while supporting healing processes. However, in individuals with psoriasis, IL-22 is believed to promote keratinocyte proliferation, leading to enhanced keratinization and the development of psoriatic lesions (94, 154, 155). Neutrophils also secrete IL-22 in psoriatic lesions, and normally IL-22 increases the proliferation of keratin-forming cells (105). The actions of IL-22 are mediated through the transmembrane receptor complex comprising IL-22R1 and IL-10R2, with IL-22R1 likely playing a significant role in the pathogenesis or exacerbation of psoriasis (156). IL-20 and IL-24 are recognized as receptor subunits of IL-22R1 (157, 158). Additionally, cytokines such as IL-17, TNF, and IL-1β can enhance the effects of IL-22. Targeting the interactions between IL-22R1 and cytokines such as IL-20, IL-24, IL-17, TNF, and IL-1β could improve clinical outcomes in psoriasis treatment (159).

#### 5.4.4 TNF-α

**Figure 4:**
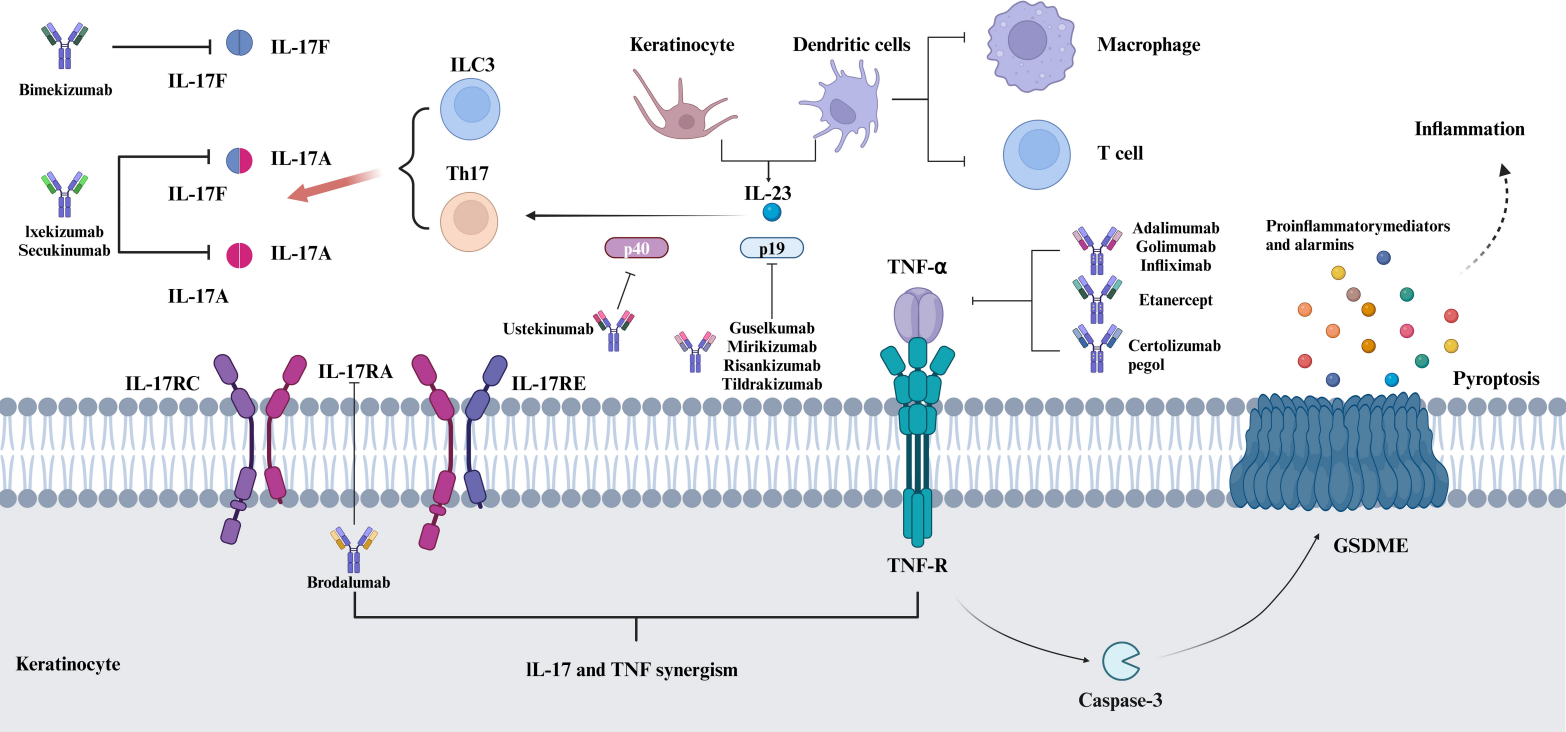
IL-17 and TNF-α: Key Psoriasis inflammation and therapeutic targets. Both IL-17 and TNF-α are key inflammatory mediators in psoriasis, and they are involved in the regulation of skin inflammation and immune responses. Both interact in the pathogenesis of psoriasis and together contribute to the inflammatory response and the development of skin lesions.IL-17 induces keratinocytes and other cell types to produce a variety of inflammatory factors, including TNF-α, which amplify the inflammatory response. Because of their role in psoriasis, IL-17 and TNF-α have become important targets for psoriasis treatment, and by inhibiting the activity of these cytokines, the symptoms of psoriasis can be effectively controlled. Biological agents targeting TNF-α and IL-17 have been widely used in the treatment of psoriasis.

TNF is a trimeric cytokine released by immune and epithelial cells during infections or tissue damage, interacting with two specific receptors: TNF receptor 1 (TNFR1) and TNFR2 (160). While TNFR1 is widely distributed across various cell types, TNFR2 exhibits high expression levels primarily in myeloid cells and lower levels in other tissues (161). The expression of TNFR2 can be upregulated by several factors, including IL-33, IFN-γ, IL-1, TL1A, and TNF itself (162). TNF, present in both membrane-bound and soluble forms, can bind to TNFR1 and TNFR2, with the membrane-bound form displaying a stronger affinity for TNFR2 (163). TNF-α, located on chromosome 6, is an important transmembrane protein with pro-inflammatory and immunomodulatory functions. TNF-α signals through two receptors, with TNFR1, also known as p55 or CD120a, being the primary receptor for TNF-α (164). The binding of TNF-α to TNFR1 is essential for regulating inflammatory signaling pathways and apoptosis. TNFR1 activates kinases including IκBα kinase-2 (IKK2), TBK1, and IKKϵ, initiating the NF-κB signaling pathway. This process triggers inflammatory cytokines such as TNF and IL-17, which initiate further inflammatory responses (**Figure 4**) (165). Unlike TNFR1, TNFR2 (also referred to as p75 or CD120b) has a lower affinity for TNF-α, yet it is capable of binding under specific conditions. Currently, TNF inhibitors primarily function by targeting TNFR1 (166). Five anti-TNF-α medications have been approved: infliximab (Remicade), adalimumab (Humira), golimumab (Simponi), certolizumab pegol (Cimzia), and etanercept (Enbrel) (167). Remicade, Humira, Simponi, and Cimzia manage autoimmune conditions like inflammatory bowel disease and psoriasis by inhibiting TNF-α and TNFR1 interaction, thereby moderating inflammation and modulating immune responses. Unlike other TNF inhibitors, Enbrel has a high affinity for TNFR2. It alleviates inflammation and immune responses by binding to and neutralizing free TNF-α in the body, and it is leveraged for various autoimmune conditions, including PsA and plaque psoriasis.

#### 5.4.5 IFN-γ

IFN-γ, an essential cytokine in the interferon family, is mainly produced by diverse immune cells, including T lymphocytes, NK cells, and macrophages. IFN-γ binds to its receptor, activating the JAK-STAT pathway and inducing the expression of responsive genes. This cytokine participates in modulating immune cell function and in initiating immune responses, making it essential for both innate and adaptive immunity. Consequently, dysregulated IFN-γ expression is strongly linked to the pathophysiology of assorted human illnesses (168, 169).

Recent studies have shown elevated levels of IFN-γ in psoriasis, with significant secretion primarily from T cells, including Th1, Th17, and Th22 subsets (63, 170). Moreover, the levels of IFN-γ in psoriatic lesions correlate closely with disease severity (170, 171). Notably, IFN-γ produced by CD4^+^ T cells has been recognized as a major contributor to the emergence of guttate psoriasis, while IFN-γ from CD8^+^ T cells appears to be more influential in the immunopathogenesis of plaque psoriasis (36). RNA sequencing and transcriptomic analyses of skin biopsies reveal increased IFN-γ signaling in non-pustular palmoplantar psoriasis, while palmoplantar pustular psoriasis is more associated with the IL-17 pathway and neutrophil-related genes like IL-36A (172). These findings suggest that IFN-γ levels may act as a biomarker for differentiating psoriasis subtypes and highlight its necessary role in the disease’s pathophysiology. Recent studies have demonstrated that IFN-γ can induce significant transcriptomic changes, such as the downregulation of miR-149, which increases KC sensitivity to the inflammatory cytokine TWEAK, thus exacerbating skin inflammation (173). Additionally, IFN-γ has been shown to modulate the activation and function of multiple immune cell populations, including T cells, macrophages, and DCs. For example, IFN-γ facilitates the transformation of human monocytes into inflammatory macrophages (174). In DCs, the inhibition of the STAT1/NF-κB signaling pathway by IFN-γ results in reduced production of pro-inflammatory cytokines and chemokines, including TNF, IL-1β, IFN-γ, and IL-17. This process impairs the development of effector CD8^+^ T cells, thereby reducing skin thickening and alleviating psoriatic symptoms like scaling and fissuring (175). These findings suggest that the downregulation of the JAK-STAT inflammatory signaling pathway downstream of IFN-γ may improve psoriasis-like lesions (175, 176).*In vitro* studies have shown that IFN-γ enhances the production of heat shock proteins HSP90α and HSP90β in HaCaT cells. Inhibition of IFN-γ resulted in alterations in the immune cell population and led to marked improvement in psoriatic lesions and symptoms (177). In summary, while the precise mechanisms of IFN-γ in psoriasis remain to be fully elucidated, current evidence underscores its involvement in the inflammatory response, abnormal KCs differentiation, and immune cell activation, revealing a critical regulatory role in the disease’s development and progression. Targeting IFN-γ and its downstream pathways could provide a promising therapeutic approach for future psoriasis management.

## 6 Other inflammatory factors

### 6.1 TGF-β

TGF-β exists in various isoforms in humans and participates in regulating physiological and pathological processes. These encompass development, immune system regulation, cell proliferation and differentiation, apoptosis, cell migration, and tissue repair (178). TGF-β dysregulation is linked to the onset and advancement of diseases such as cancer, inflammatory disorders, and fibrosis. It is widely acknowledged that psoriatic lesions exhibit downregulated levels of TGF-β, which typically functions to inhibit KC growth; thus, a decrease in TGF-β concentrations within the skin can facilitate KC uncontrolled proliferation. Researches reveal that Growth Differentiation Factor 11 (GDF11), part of the TGF-β superfamily, eases skin inflammation in a mouse model of psoriasis induced by imiquimod. GDF11 administration significantly improved symptoms like erythema, desquamation, and epidermal thickening, while reducing inflammatory cell infiltration, epithelial hyperplasia, and levels of inflammatory mediators such as IL-1β, TNFα, COX-2, iNOS, and IL-6 (179). Infusion of mouse mesenchymal stromal cells that secrete IL-6 and TGF-β has been illustrated to accelerate the resolution of psoriasis-like skin inflammation, marked by epidermal thinning and reduced CD3^+^ T cell infiltration. The upregulation of TGF-β and IL-17A in the skin likely inhibits T cell-mediated pathological processes (180). In contrast to previous findings, certain studies suggest a positive correlation between TGF-β levels and psoriasis development. In particular, TGF-β1 is significantly upregulated in psoriatic KCs and promotes the overexpression of keratin 17 and the proliferation of KCs through the TGF-β/SMAD/miR-486-3p signaling axis, thereby promoting the development of psoriasis (181). Similarly, GDF15, part of the TGF-β superfamily, presents decreased expression in the epidermis of psoriasis patients and animal models. It suppresses cytokine and chemokine production by keratinocytes and directly inhibits neutrophil adhesion and migration, spotlighting its anti-inflammatory effects that ameliorate psoriasis (182). In conclusion, TGF-β emerges as a crucial biological regulator that profoundly influences cellular and tissue functionality, alongside the onset and progression of diseases. However, elucidating the precise role of TGF-β in psoriasis, along with the specific mechanisms and regulatory networks involved, necessitates further investigation to achieve a comprehensive understanding.

### 6.2 CXCLs

**Figure 5:**
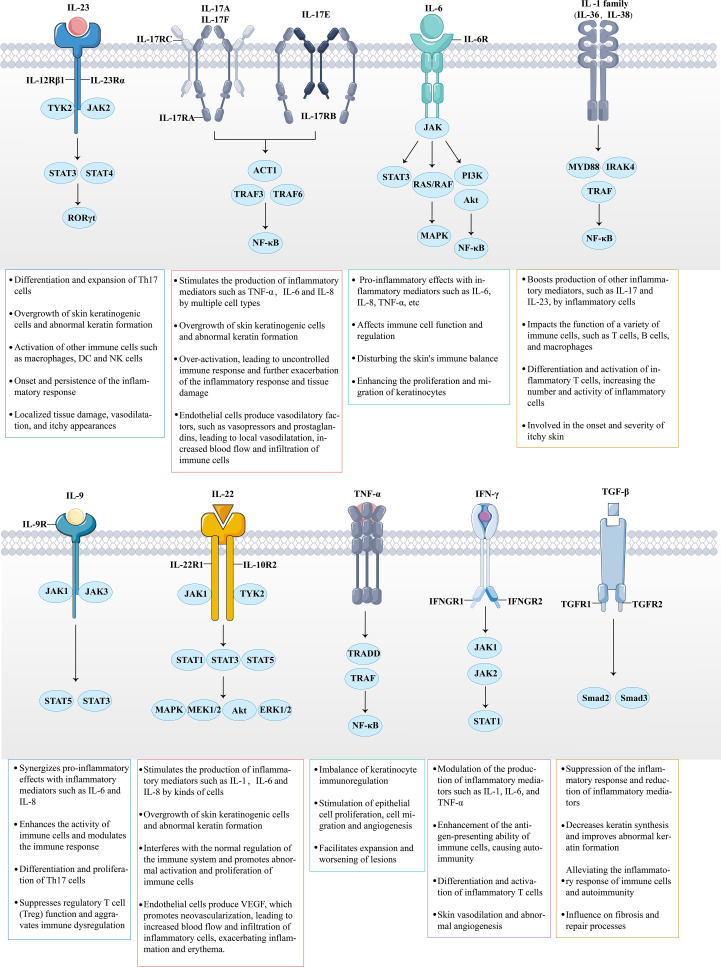
The role of multiple cytokines and signaling pathways in psoriasis is highly significant. Cytokines secreted by immune cells bind to their corresponding receptors. For instance, the massive release of IL-23, IL-6, IL-9, IL-22, and IFN-γ activates the intracellular JAK-STAT signaling pathway, thereby triggering downstream inflammatory responses. Meanwhile, IL-17, members of the IL-1 family, and TNF-α amplify the inflammatory response by activating the NF-κB signaling pathway. Under the combined influence of these inflammatory factors and signaling pathways, the inflammatory response in psoriasis continues to escalate, promoting the involvement of additional inflammatory cells (such as Th17 and DCs). This results in a multidimensional stimulation of keratinocytes, ultimately leading to further deterioration of the psoriasis condition.

Chemokines represent a specific category of cytokines that direct and activate the movement of immune cells and other cell types towards particular chemotactic gradients, thereby playing an essential role in immune system function. Abnormal chemokine expression during disease processes can initiate and exacerbate inflammatory disorders such as psoriasis. This study explores the regulatory roles and mechanisms of chemokines CXCL9, CXCL10, and CXCL11 in psoriasis. Multiple chemokines have higher expression levels within the lesion-prone skin of psoriasis sufferers, including the CXC chemokine family members CXCL9, CXCL10, and CXCL11, with increased expression of CXCL9/10 being characteristic of psoriasis (183). CXCL10 is suggested as a potential biomarker for psoriasis progression, but existing evidence is inadequate to confirm this, necessitating further validation (184). The effects of CXCL9, CXCL10, and CXCL11 are mediated through their specific receptor, CXCR3, with CXCL11 recognized as the primary agonist due to its superior chemotactic potency relative to CXCL10 and CXCL9 (185). CXCR3 is predominantly expressed on monocytes, endothelial cells, fibroblasts, and cancer cells. Activation of CXCR3 has been demonstrated to attract CD4^+^ T lymphocytes and CD8^+^ T lymphocytes to inflammatory sites while also facilitating their differentiation into more effective effector T cells (185). The accumulation of immune cells in affected areas can trigger inflammatory responses and subsequent tissue damage. These inflammatory processes, coupled with immune cell activation, further stimulate keratinization, leading to the abnormal growth and differentiation of skin cells that manifest as the characteristic scales and lesions of psoriasis. Endothelial cells, fibroblasts, and KCs release CXCL9, CXCL10, and CXCL11 in response to IFN-γ, with CXCL10 significantly influencing Th1 cell development and function (186, 187). In tissues, recruited Th1 cells can increase IFN-γ and TNF-α secretion, which in turn stimulates CXCL10 production by various cells, creating a feedback loop that sustains autoimmune processes (186, 187). Recent research suggests that estrogen affects the expression of immune cells (DCs and γδT cells), chemokines (CCL5 and CXCL10), and cytokines (TNF-α, IL-23, and IL-17 family), which worsens cutaneous inflammation in psoriasis and provides new insights for developing treatment strategies (188). It should be noted that psoriasis is a multifaceted disease involving multiple cytokines and signaling pathways in its development and progression (**Figure 5**). Chemokines constitute one facet of this complexity, contributing to the disease’s etiology by attracting and regulating the migration and activation of immune cells, promoting inflammatory responses, and interacting with various factors to collectively influence pathological processes. In-depth investigation of chemokine mechanisms is essential for enhancing our understanding of psoriasis pathogenesis and developing novel treatments.

## 7 Drugs targeting cytokines for psoriasis

Psoriasis pathogenesis is regulated by various immune cells and cytokines. As research in this area continues, treatments are becoming more diverse. Conventional therapies for psoriasis employ a wide range of anti-inflammatory and immunosuppressive treatments. These include topical corticosteroids for localized disease, as well as NSAIDs, vitamin D analogues (e.g., calcipotriol), and vitamin A analogues. Phototherapy options, such as broadband UVB, narrowband UVB, PUVA, and tanning, are also utilized. For refractory or extensive psoriasis, systemic therapies like methotrexate (MTX), retinoic acid, and cyclosporine are commonly prescribed (11, 189). However, these treatments have limited efficacy and may cause adverse reactions. They are also not suitable for most patients. UVB treatment reduces the infiltration of p38 pY323^+^ T cells in psoriatic lesions and suppresses IRF4 and IL-17 production, which may contribute to the therapeutic effects of phototherapy in psoriasis (190). UVB also induces CD95L expression in keratinocytes, leading to intradermal T-cell depletion. Additionally, it influences psoriasis manifestation by promoting vitamin D production and modulating immune responses to local antigens. However, prolonged use of UVB therapy increases the risk of photoaging and skin cancer (191, 192). Although MTX is a first-line treatment for moderate-to-severe psoriasis and PsA, its long-term use is limited by gastrointestinal toxicity, myelosuppression, and hepatic and pulmonary injury. For mild PsA cases, short-term use of nonsteroidal anti-inflammatory drugs may be appropriate, whereas oral glucocorticoids are not recommended (193, 194). Topical corticosteroids are often considered skin sensitizers, and prolonged use may cause adverse effects such as skin atrophy, osteoporosis, and contact dermatitis (195–197). In contrast, biologic therapies represent a new paradigm of targeted treatment modalities that offer enhanced safety, improved effectiveness, and a reduced incidence of side effects. These biologics exert their therapeutic effects by specifically modulating pivotal cytokines implicated in the immunopathogenesis of psoriasis (198). This article aims to systematically summarize and thoroughly review advancements in drug development strategies that target key cytokines associated with psoriasis. Our objective is to provide valuable insights and references to inform future endeavors in the creation of specific therapeutic agents for psoriasis (**Table 1**).

**Table 1:** Cytokine-based therapeutic drugs and clinical research.

Cytokines	Function	Drugs	Indications	Long-term outcomes	Side effects	Phase	NCT
IL-23	Promotes the production of inflammatory cytokines by Th17 cells, leading to activation and hyperproliferation of keratin-forming cells.	Tildrakizumab	Adult patients with moderate to severe plaque psoriasis	Drugs are safe and effective in tests measuring their effects on skin and blood immune cells in psoriasis patients Safe and effective for treating PsA	Acute myocardial infarction, COVID-19 pneumonia, deep vein thrombosis, hypertension, nasopharyngitis, pharyngitis, rhinitis, urinary tract infection, upper respiratory tract infection	Marketing	NCT05390515 NCT03552276
		Guselkumab	Adult patients with moderate to severe plaque psoriasis	There is good security and the rate of security incidents remains stable.	Nasopharyngitis, upper respiratory tract infection, rectal adenocarcinoma, prostate cancer, plasma cell myeloma, melanoma *in situ*, nonfatal myocardial infarctions, nonfatal stroke and uveitis	Marketing	NCT02207231 NCT02203032 NCT02905331
		Risankizumab	Crohn’s disease, psoriatic arthritis and plaque psoriasis	Risankizumab consistently demonstrated higher efficacy compared to sukinumab in different patient groups. High induction doses of risankizumab were effective in patients with moderate to severe plaque psoriasis.	Nasopharyngitis, upper respiratory tract infection, oral or vulvovaginal candida infections	Marketing	NCT03478787 NCT05283135
		IBI112 (Picankibart)	Moderate to severe plaque psoriasis	Picankibart has a significant short-term onset of action, superior efficacy, and a favorable safety profile.	Not Available	Phase 3	NCT06049810 NCT05645627
		Mirikizumab	Plaque psoriasis	Mirikizumab shows superior efficacy to placebo in treating moderate to severe plaque psoriasis. Overall safety and efficacy were assessed by long-term mirikizumab treatment.	Injury, poisoning and procedural complications, cerebral infarction, basal cell carcinoma, eczema, allergic rhinitis, injection-site pain, hypertension and diarrhoea	Marketing	NCT03718884 NCT03482011 NCT02899988
IL-17	Stimulate epidermal hyperplasia and the pro-inflammatory feed-forward loop seen in plaque psoriasis	Secukinumab	Adults with moderate-to-severe plaque psoriasis and pediatric patients	By curing mild psoriasis with short-term anti-IL-17A therapy, investigators could reduce the cost of treating psoriasis and related diseases, including psoriatic arthritis, cardiovascular disease, and diabetes. Risankizumab consistently demonstrated higher efficacy compared to sukinumab in different patient groups	Nasopharyngitis, upper respiratory tract infection, oral or vulvovaginal candida infections, inflammatory bowel disease (ulcerative colitis)	Marketing	NCT03131570 NCT03478787 NCT02592018
		Ixekizumab	Adult patients with moderate to severe plaque psoriasis	Ixekizumab delivery concentrations explored, paving the way for personalized treatment	Not Available	Marketing	NCT02993471 NCT04083612
		Brodalumab	Moderate to severe psoriasis	Brodalumab treatment produced significant clinical benefits and an acceptable safety profile in patients with moderate to severe plaque psoriasis.	Nasopharyngitis, upper respiratory tract infection, headache, depression, Candida infection and arthralgia.	Marketing	NCT01708590 NCT01708603
		Bimekizumab	Adults with moderate-to-severe plaque psoriasis, generalized pustular psoriasis, and erythrodermic psoriasis	Bimekizumab was well tolerated with no unexpected safety findings.	Upper respiratory tract infection, nasopharyngitis, viral meningitis, colorectal polyps, colon cancer and fungal infections (oral candidiasis, oral fungal infections, vulvovaginal fungal infections and tinea pedis)	Marketing	NCT03025542 NCT02905006 NCT03010527
		Netakimab (BCD-057)	Moderate to severe plaque psoriasis	BCD-057 was well tolerated and comparable to iADA in terms of efficacy, IG, steady-state PK, and safety, with no significant treatment switching effects.	Toxic dermatosis, post-traumatic osteoarthritis of the interphalangeal joints, chickenpox, head injury resulting in death, acute bilateral suppurative maxillary sinusitis	Marketing	NCT02762955
		Vunakizumab (SHR-1314)	Ankylosing spondylitis, plaque psoriasis, Graves’ ophthalmopathy	SHR-1314 is superior in subjects with moderate to severe plaque psoriasis.	Upper respiratory tract infections and hyperuricemia	Phase 1/2	NCT03463187 NCT02934412
		Izokibep (ABY-035)	Moderate to severe plaque psoriasis	ABY-035 demonstrated good efficacy, safety and tolerability in different dose groups and administration frequencies	Itching and pain	Phase 1 Phase 2	NCT03580278 NCT03591887
		ZL1102	Chronic plaque psoriasis	ZL-1102 developed specifically for the treatment of mild to moderate chronic plaque psoriasis.	Not Available	Phase 2	NCT06380907
IL-36	Acts on keratinocytes, epithelial cells, and immune cells, causing cytokine secretion and excessive inflammatory responses and aggregation of immune cells	ANB019 (AnaptysBio)	Generalized pustular psoriasis in adults	ANB019 showed a favorable safety and tolerability profile in patients with generalized pustular psoriasis.	Not Available	Phase 2 Phase 3	NCT03619902 NCT05352893 NCT03633396
		Spesolimab	Generalized Pustular Psoriasis	Spesolimab provides rapid and sustained clinical improvement in efficacy and safety by delivering GPP-targeted therapy.	Urinary tract infections, influenza, folliculitis, otitis externa, upper respiratory tract infections, pustules, weakness, fatigue, nausea, vomiting, headache, itching, and infusion site hematoma and bruising	Phase 1, Phase 3	NCT02978690 NCT05200247 NCT05239039
IL-12	Induces the production of IFN-γ and others through multiple pathways, affecting the activation and proliferation of keratinocytes.	Ustekinumab	Moderate-to-severe plaque psoriasis in adults	Ustekinumab shows a favorable safety profile. Ustekinumab is well tolerated and effective in patients with inadequate methotrexate response.	Headaches, nasopharyngitis, joint pain, high blood pressure and itching	Marketing	NCT00307437 NCT00267969 NCT01059773
		Briakinumab	Moderate to severe plaque psoriasis	Results of a Pharmacokinetic evaluation of single-dose CYP substrate by brenezumab in subjects with moderate to severe psoriasis.	Not Available	Withdrawn	NCT01260844
TNF-α	Induces the production of other cytokines, promoting vasodilatation and increasing permeability, which leads to the infiltration of inflammatory cells and the formation of inflammatory cell-mediated lesions, but also promotes the aberrant proliferation and hyperkeratosis of keratinocytes	Infliximab	Moderate-to-severe plaque psoriasis in adults	Infliximab superior to placebo for palmoplantar psoriasis.	Cellulitis of the cheeks, hepatitis, fatigue, nausea and loss of appetite, jaundice	Marketing	NCT00629772 NCT00261976
		Adalimumab	Moderate to severe plaque psoriasis in adults, severe plaque psoriasis in children	Lung infections, pneumonia and tuberculosis, fever, tachycardia and asthma-like symptoms	The duration of the treatment period was not sufficient to assess long-term efficacy and safety, and the trial did not include an active control consisting of other systemic therapies.	Marketing	NCT03217734 NCT01646073
		Etanercept	Moderate to severe plaque psoriasis in adults	Patients from six countries in Asia, Central Europe and Latin America responded to etanercept treatment similarly to those observed in patients in the United States and Western Europe.	Nasopharyngitis, headache, elevated blood insulin, diarrhea, injection-site erythema, pharyngitis, arthralgia, injection-site reaction, fatigue, rash, nausea, anemia, mucositis, neutropenia, respiratory distress, lung infections and sepsis	Marketing	NCT00663052 NCT02258282
		Certolizumab	Moderate to severe plaque psoriasis in adults	CZP has long-term clinical efficacy in patients with moderate to severe plaque psoriasis. Clinical signs and symptoms of PsA improved after CZP treatment.	Nasopharyngitis, upper respiratory tract infection, hypertension, mesenchymal oligodendroglioma, basal cell carcinoma, breast cancer, clear cell renal carcinoma, glioblastoma, Hodgkin’s disease, keratoacanthoma, diarrhea, headache and laryngeal carcinoma	Marketing	NCT02346240 NCT02326298 NCT01087788

### 7.1 IL-23 inhibitors

In the pathophysiology of psoriasis, IL-23 performs a crucial upstream regulatory position by encouraging Th17 cell activation, which results in the secretion of a variety of inflammatory cytokines. By activating and proliferating KCs, these types of cytokines collaborate in concert to exacerbate psoriatic skin disorders (60). Notably, the inhibition of IL-23 does not interfere with the normal physiological roles of other cytokines, such as IL-17, derived from different cellular sources, making IL-23 a highly attractive therapeutic target for psoriasis. Approved by the FDA in 2017, Guselkumab is the first biologic agent targeting IL-23 for treating moderate-to-severe plaque psoriasis in adults (199). Due to its enhanced safety and effectiveness, its application has been extended for treating PsA (200). Tildrakizumab, a humanized antibody with high affinity for the IL-23 p19 subunit, received FDA approval in 2018 for treating moderate-to-severe plaque psoriasis in adults. A Phase 3 clinical trial among Chinese patients confirmed its strong efficacy and safety in treating moderate-to-severe plaque psoriasis (201). Approved in 2019, Risankizumab is the third FDA-approved inhibitor of the IL-23 p19 subunit for treating moderate-to-severe plaque psoriasis in adults, demonstrating a favorable safety and tolerability profile (202). Risankizumab, approved in Japan for generalized pustular psoriasis, erythrodermic psoriasis, and PsA, also demonstrates potential for treating autoimmune diseases worldwide, including hidradenitis suppurativa, ulcerative colitis, Crohn’s disease, asthma, atopic dermatitis, and ankylosing spondylitis (203). Mirikizumab, the latest IL-23 inhibitor to be approved by the FDA in October 2023, focuses on inflammatory bowel disease rather than psoriasis in its approved indication. However, clinical evidence has demonstrated the efficacy of Mirikizumab in psoriasis, providing significant relief of psoriatic lesions, thereby underscoring its potential as a prominent treatment option for the condition (204, 205). Beyond the four FDA-approved IL-23 inhibitors, numerous other biologic agents targeting IL-23 are in clinical trials, underlining the potential of IL-23 as a therapeutic target for psoriasis.

### 7.2 IL-17 inhibitors

The IL-17 family, particularly IL-17A, plays a pivotal role in psoriasis by acting as a pro-inflammatory factor that induces the expression of chemokines and cytokines, recruits neutrophils and monocytes, and drives inflammation (79). The prominent role of the IL-17 pathway in psoriasis has led to blocking IL-17 signaling becoming a vital strategy for treating psoriasis. Presently, drug development for this purpose focuses on two primary strategies: targeting anti-IL-17A and IL-17RA. In 2015, the FDA approved Secukinumab, a fully human monoclonal antibody targeting IL-17A, for adults with moderate-to-severe plaque psoriasis unsuitable for phototherapy or systemic therapy. Secukinumab specifically targets IL-17A epitopes, acts quickly, is well-tolerated with few side effects, and minimizes anti-drug antibody production (206, 207). Similarly, Ixekizumab, another humanized monoclonal antibody targeting IL-17A, received FDA approval in 2016 based on its robust efficacy and safety profile, and has been widely marketed (208). Netakimab, a chimeric IgG1 monoclonal antibody against IL-17A, was developed in Russia and approved in 2019 for moderate-to-severe plaque psoriasis. In 2020, its utilization was extended to ankylosing spondylitis and PsA. Clinical trials are ongoing in the U.S. and China (209). Bimekizumab, a monoclonal antibody that targets IL-17A and IL-17F, received marketing approval in Europe in 2021 and in Japan in 2022 for the treatment of psoriasis and PsA (209). Unlike the four aforementioned drugs, Brodalumab, a humanized IgG2 monoclonal antibody targeting IL-17RA, has predominantly been utilized for the intervention of same indications since its approval in Europe in 2017 (210). Recent multicenter researches have confirmed Brodalumab’s efficacy and safety in both short-term and long-term psoriasis treatment (211). In addition to these five FDA-approved drugs, ongoing research and development efforts are exploring additional IL-17-targeting therapies, such as Vunakizumab, Izokibep (ABY-035), and ZL1102, all of which show promising potential for psoriasis treatment.

### 7.3 IL-36 inhibitors

Continuous abnormal activation of IL-36R leads to the production of chemokines and cytokines, which in turn encourages the infiltration and activation of different inflammatory cells such as macrophages, neutrophils, and Th17 cells. This activation induces cytokine release, including IL-17, TNF, and IL-22, which in turn enhances IL-36 expression by skin cells and DCs. Consequently, a feedback loop intensifies the inflammatory response, attracting numerous neutrophils and other immune cells to the site. This process leads to the formation of pustular patches and the onset of pustular psoriasis (123, 124). Therefore, the drugs currently undergoing clinical studies primarily target IL-36R. The main examples include Imsidolimab (ANB019) and Spesolimab.

### 7.4 IL-12 inhibitors

IL-12 and IL-23 are part of the same family, both containing a shared p40 subunit, while IL-12 uniquely includes a p35 subunit. The pathogenesis of psoriasis is influenced by IL-12/23, making it a crucial target for blocking the development of the condition (60). In 2009, the FDA approved Ustekinumab, a monoclonal antibody targeting the IL-12/IL-23 p40 subunit, for treating moderate-to-severe plaque psoriasis in adults (212). In recent years, its indications have expanded to include PsA and ulcerative colitis. Common adverse effects of Ustekinumab include mild nasopharyngitis and headaches; nonetheless, it generally exhibits a favorable safety profile (212).

### 7.5 TNF-α inhibitors

TNF-α, a key cytokine linking inflammation and immune responses, is released by activated macrophages, NK cells, and T lymphocytes. Its biological functions, including cell proliferation, metabolic activation, and inflammatory response, are substantial in psoriasis pathogenesis (213). The four approved and marketed drugs targeting TNF-α are Infliximab, Adalimumab, Etanercept, and Certolizumab. Their main indications include psoriasis, ankylosing spondylitis, inflammatory bowel disease, and other autoimmune diseases. However, the safety of TNF-α inhibitors requires further improvement. Studies have revealed that some female patients treated with TNF-α monoclonal antibodies for inflammatory bowel disease developed psoriasis, with the underlying mechanism remaining obscure and warranting further investigation (214). The core mechanism of action of the characteristic metabolite polycyclic polypentadienylacylated acyl resorcinols (PPAPs) from Hypericum perforatum includes selective inhibition of the TNF-α signaling pathway, which alleviates psoriasis-associated pathologic epidermal thickening through modulation of the epidermal cell proliferation cycle, and the compounds provide potential candidate molecules for psoriasis treatment through multi-targeted intervention in the TNF-α-associated signaling network (215).

### 7.6 Biomarkers for predicting response to therapy

There is currently no cure for psoriasis or PsA, however, biologic therapies targeting key inflammatory pathways have demonstrated significant efficacy. Identifying reliable biomarkers is crucial for predicting disease prognosis and treatment response, thereby aiding in the optimization of personalized therapeutic strategies. The primary biomarker categories identified to date include genetic, blood-based, tissue-derived, and transcriptional markers (216). Studies indicate that the response to anti-IL-17 therapy may be influenced by common genetic variants, HLA alleles, polygenic risk scores, and shared genetic susceptibility across multiple diseases (217). The key gene PYCARD, primarily involved in inflammatory pathways, is highly expressed in keratinocytes and significantly upregulated in psoriatic lesions. Studies have demonstrated its crucial role in the development of psoriatic lesions (218). The ABCC1 and ABCG2 SNPs are associated with a favorable response to methotrexate in psoriasis patients, while DHFR SNPs show similar effects in PsA. In PsA patients, the TNF gene promoter -308 G/G genotype exhibits greater responsiveness to anti-TNF therapy. After anti-TNF treatment, Th1 and Th17 cell frequencies decrease in psoriasis patients, reinforcing the critical role of the IL-23/Th17 axis in disease pathology. Additionally, IL-23, IL-23R, and Th17-related cytokines are elevated in psoriatic lesions compared to non-lesional skin. This upregulation is accompanied by increased expression of anti-apoptotic proteins (Bcl-X, Bax, and Bak), which correlate with response to anthracycline and anti-TNF therapy (219). The immune response in psoriasis involves inflammatory mediators that are strongly linked to vascular inflammation and the progression of atherosclerosis. Consequently, atherosclerosis-related biomarkers may also serve as therapeutic indicators for psoriasis (220). Deucravacitinib modulates inflammatory cytokines, including IL-23 and type I interferon, by targeting the Tyk2 signaling pathway. Additionally, it influences biomarkers related to collagen matrix turnover, which may serve as predictors of its therapeutic response in PsA (221). TNF-α is a key therapeutic target for PsA and has shown significant efficacy in clinical trials. Several proteins are crucial in inflammation regulation and immune response, including apolipoproteins, fibrinogen, type II collagen, serum amyloid A, conjugated bead proteins, 14-3-3 proteins, and S100 family proteins, particularly S100A8. Additionally, major drug-binding proteins such as human serum albumin and α-1-acid glycoprotein have been identified as potential predictors of response to anti-TNF-α therapy in PsA. Changes in their levels may be linked to TNF-α neutralization (222). Studies have shown that golimumab regulates acute-phase reactants, inflammatory markers, metabolic factors, and bone remodeling markers. Serum levels of CRP, VEGF, MMP-3, and ICAM-1 are strongly correlated with psoriasis-related scores, and some of these markers have been linked to clinical endpoints of anti-TNF therapy in RA studies. Additionally, IL-6, VEGF, MMP-3, and YKL-40 levels are significantly associated with the response to anti-TNFα therapy in patients with spondyloarthropathies (223).

### 7.7 Others

In addition to those previously noted, cytokines like IFN-γ, TGF-β, CXCL9, CXCL10, IL-33 and CXCL11 play a significant role in psoriasis pathogenesis. In addition, natural compounds can also effectively regulate the expression of cytokines. For example, natural products (e.g., the flavonoid tetramethoxy lignans/methlut) can inhibit IL-33 production and its signaling with the ST2 receptor; and reduce downstream inflammatory factor release by inhibiting IL-33/Substance P-mediated mast cell activation. It is a preferred lead compound for the development of drugs for the treatment of psoriasis (224). Curcumin inhibits psoriasis inflammation and keratinocyte proliferation through downregulation of pro-inflammatory cytokines and inhibition of IL17/IL23, NF-κB and STAT-3 signaling pathway activities (225, 226). However, their transdermal absorption is limited, compromising efficacy. To improve delivery efficiency, nanoparticles are used as curcumin carriers (216). Common formulations include nanosuspensions, nanoparticles, nanoemulsions, solid lipid particles, nanocapsules, nanospheres and liposomes (227).

It is crucial to acknowledge that, despite the continuous development of various therapeutic agents for psoriasis, the overall non-response rate to TNF-α inhibitors remains significantly high, exceeding 30% (228). Furthermore, approximately 10% to 20% of patients do not respond to biologics targeting IL-17 and IL-23 (206, 229, 230). The reasons for this lack of efficacy are multifactorial and may encompass several key factors: (1) Although recent studies have emphasized the predominant role of the Th17 pathway in mediating inflammation in psoriasis and psoriatic arthritis, other signaling pathways, such as TNF-α and IFN-γ, are also critically important (1). Therefore, reliance on a single IL-17, IL-23 and TNF-α inhibitor may not sufficiently address the diverse critical pathways involved in psoriasis, potentially resulting in suboptimal outcomes in targeted therapy. (2) Additionally, the IL-17 family comprises several members beyond IL-17A, with evidence indicating that IL-17F plays a significant role in the pathogenesis of psoriasis (1). Further research is warranted to explore the potential involvement of other IL-17 family members in similar mechanisms. (3) Moreover, polymorphisms within the IL-23 gene have been identified (231), suggesting that variants in the IL-23R gene may diminish the efficacy of currently available IL-23 inhibitors. (4) Recent studies have also increasingly highlighted a strong correlation between gut microbiota and the immune imbalance of T-cells in psoriasis, offering new insights into the disease’s underlying mechanisms (232–234).

Clinical trials are currently underway for drugs that target cytokines, and it is anticipated that these drugs will address the limitations of existing treatments and exhibit improved therapeutic effects. The introduction of these drugs holds the potential for revolutionary advancements in the future management of psoriasis.

## 8 Summary and prospects

Over recent decades, significant advancements have been made in understanding the intricate cytokine networks involved in psoriasis. These findings have significantly enhanced our understanding of psoriasis pathophysiology and laid a strong foundation for developing novel therapies, providing renewed hope for patients. The pathogenesis of psoriasis is driven by a complex interplay of cytokines, which collectively modulate immune responses and inflammatory processes through intricate positive and negative feedback loops. In particular, targeted therapeutic strategies aimed at these key cytokines have proven effective in controlling the abnormal immune response in psoriasis. Recognizing the pleiotropic and context-dependent roles of cytokines in psoriasis immunity is vitally indispensable for a comprehensive understanding. To achieve personalized and precise treatment, the development of biomarkers that can predict patients’ responses to specific cytokine-targeted therapies is crucial. This will help doctors tailor the most appropriate treatment plan for each patient, thereby maximizing therapeutic effects. The future of psoriasis therapy is evolving beyond monoclonal antibodies to include small-molecule inhibitors, gene-editing technologies, RNA-based therapies, and nanocarriers (235). Oral inhibitors like JAK-STAT (e.g., deucravacitinib) and PI3K/NF-κB blockers offer convenience and potential for cytokine modulation, though specificity and long-term safety remain challenges. Gene-editing tools such as CRISPR-Cas9 can precisely target cytokine-related genes (e.g., silencing IL-23R), with ex vivo T cell or stem cell editing promising personalized treatments. RNA therapies targeting IL-17C, IL-36γ, or TNF-α can suppress inflammation, boosted by nanoparticle delivery for stability and skin penetration. Engineered Tregs and CAR-T cells aim to restore immune balance by targeting Th17 activity or pathogenic cells. Nanocarriers, like curcumin-loaded particles, enhance topical drug delivery while minimizing systemic exposure. As biomedical research advances, a multimodal strategy integrating these innovations will drive precise, personalized treatments and improve outcomes for psoriasis patients. Furthermore, combining cytokine-targeted therapies with emerging CAR-T therapies and phototherapy may provide a more comprehensive treatment strategy for psoriasis patients. This integrated approach is expected to improve efficacy while reducing adverse effects and enhancing life satisfaction in patients. As biomedical research progresses, the treatment of psoriasis in the future will become more precise and personalized, offering a brighter outlook for patients.

## References

[1] Griffiths CEM Armstrong AW Gudjonsson JE Barker J. Psoriasis. Lancet. (2021) 397:1301–15. doi: 10.1016/s0140-6736(20)32549-6 33812489

[2] Hwang JK Grover C Iorizzo M Lebwohl MG Piraccini BM Rigopoulos DG. Nail psoriasis and nail lichen planus: Updates on diagnosis and management. J Am Acad Dermatol. (2024) 90:585–96. doi: 10.1016/j.jaad.2023.11.024 38007038

[3] Mossner R Wilsmann-Theis D Oji V Gkogkolou P Lohr S Schulz P. The genetic basis for most patients with pustular skin disease remains elusive. Br J Dermatol. (2018) 178:740–8. doi: 10.1111/bjd.15867 28887889

[4] Francis L Capon F Smith CH Haniffa M Mahil SK. Inflammatory memory in psoriasis: From remission to recurrence. J Allergy Clin Immunol. (2024) 154:42–50. doi: 10.1016/j.jaci.2024.05.008 38761994

[5] Raychaudhuri SK Maverakis E Raychaudhuri SP. Diagnosis and classification of psoriasis. Autoimmun Rev. (2014) 13:490–5. doi: 10.1016/j.autrev.2014.01.008 24434359

[6] Zhou Y Xu F Chen XY Yan BX Wang ZY Chen SQ. The epidermal immune microenvironment plays a dominant role in psoriasis development, as revealed by mass cytometry. Cell Mol Immunol. (2022) 19:1400–13. doi: 10.1038/s41423-022-00940-8 PMC9708662 36348078

[7] Yi M Li T Niu M Zhang H Wu Y Wu K. Targeting cytokine and chemokine signaling pathways for cancer therapy. Signal Transduct Target Ther. (2024) 9:176. doi: 10.1038/s41392-024-01868-3 39034318 PMC11275440

[8] Cohen C Mhaidly R Croizer H Kieffer Y Leclere R Vincent-Salomon A. WNT-dependent interaction between inflammatory fibroblasts and FOLR2+ macrophages promotes fibrosis in chronic kidney disease. Nat Commun. (2024) 15:743. doi: 10.1038/s41467-024-44886-z 38272907 PMC10810789

[9] Ghoreschi K Balato A Enerback C Sabat R. Therapeutics targeting the IL-23 and IL-17 pathway in psoriasis. Lancet. (2021) 397:754–66. doi: 10.1016/S0140-6736(21)00184-7 33515492

[10] Elnabawi YA Garshick MS Tawil M Barrett TJ Fisher EA Lo Sicco K. CCL20 in psoriasis: A potential biomarker of disease severity, inflammation, and impaired vascular health. J Am Acad Dermatol. (2021) 84:913–20. doi: 10.1016/j.jaad.2020.10.094 PMC8049184 33259876

[11] Armstrong AW Read C. Pathophysiology, clinical presentation, and treatment of psoriasis: A review. Jama. (2020) 323:1945–60. doi: 10.1001/jama.2020.4006 32427307

[12] Li L Wu X Wu J Zhang X Miao F Wang J. Transdermal delivery of Fn14 siRNA using a novel composite ionic liquid for treatment of psoriasis-like skin lesions. J Control Release. (2024) 365:818–32. doi: 10.1016/j.jconrel.2023.12.009 38070601

[13] Adachi A Honda T Egawa G Kanameishi S Takimoto R Miyake T. Estradiol suppresses psoriatic inflammation in mice by regulating neutrophil and macrophage functions. J Allergy Clin Immunol. (2022) 150:909–919 e908. doi: 10.1016/j.jaci.2022.03.028 35589416

[14] Liu C Chu D Kalantar-Zadeh K George J Young HA Liu G. Cytokines: from clinical significance to quantification. Adv Sci (Weinh). (2021) 8:e2004433. doi: 10.1002/advs.202004433 34114369 PMC8336501

[15] Cui A Huang T Li S Ma A Perez JL Sander C. Dictionary of immune responses to cytokines at single-cell resolution. Nature. (2024) 625:377–84. doi: 10.1038/s41586-023-06816-9 PMC10781646 38057668

[16] Zheng D Liwinski T Elinav E. Inflammasome activation and regulation: toward a better understanding of complex mechanisms. Cell Discovery. (2020) 6:36. doi: 10.1038/s41421-020-0167-x 32550001 PMC7280307

[17] Dong C. Cytokine regulation and function in T cells. Annu Rev Immunol. (2021) 39:51–76. doi: 10.1146/annurev-immunol-061020-053702 33428453

[18] Singh R Koppu S Perche PO Feldman SR. The cytokine mediated molecular pathophysiology of psoriasis and its clinical implications. Int J Mol Sci. (2021) 22:12793. doi: 10.3390/ijms222312793 34884596 PMC8657643

[19] Guo J Zhang H Lin W Lu L Su J Chen X. Signaling pathways and targeted therapies for psoriasis. Signal Transduct Target Ther. (2023) 8:437. doi: 10.1038/s41392-023-01655-6 38008779 PMC10679229

[20] He Y Liu T. Oxidized low-density lipoprotein regulates macrophage polarization in atherosclerosis. Int Immunopharmacol. (2023) 120:110338. doi: 10.1016/j.intimp.2023.110338 37210916

[21] Xiao Y Yang Y Xiong H Dong G. The implications of FASN in immune cell biology and related diseases. Cell Death Dis. (2024) 15:88. doi: 10.1038/s41419-024-06463-6 38272906 PMC10810964

[22] Ahmadian M Suh JM Hah N Liddle C Atkins AR Downes M. PPARγ signaling and metabolism: the good, the bad and the future. Nat Med. (2013) 19:557–66. doi: 10.1038/nm.3159 PMC3870016 23652116

[23] Sun Q Xing X Wang H Wan K Fan R Liu C. SCD1 is the critical signaling hub to mediate metabolic diseases: Mechanism and the development of its inhibitors. BioMed Pharmacother. (2024) 170:115586. doi: 10.1016/j.biopha.2023.115586 38042113

[24] Shen S Shen M Kuang L Yang K Wu S Liu X. SIRT1/SREBPs-mediated regulation of lipid metabolism. Pharmacol Res. (2024) 199:107037. doi: 10.1016/j.phrs.2023.107037 38070792

[25] Liu S Pi J Zhang Q. Signal amplification in the KEAP1-NRF2-ARE antioxidant response pathway. Redox Biol. (2022) 54:102389. doi: 10.1016/j.redox.2022.102389 35792437 PMC9287733

[26] Zhang G Wei H Zhao A Yan X Zhang X Gan J. Mitochondrial DNA leakage: underlying mechanisms and therapeutic implications in neurological disorders. J Neuroinflamm. (2025) 22:34. doi: 10.1186/s12974-025-03363-0 PMC11806845 39920753

[27] Kortekaas Krohn I Callewaert C Belasri H De Pessemier B Diez Lopez C Mortz CG. The influence of lifestyle and environmental factors on host resilience through a homeostatic skin microbiota: An EAACI Task Force Report. Allergy. (2024) 79:3269–84. doi: 10.1111/all.16378 PMC11657040 39485000

[28] Bingöl FG Ağagündüz D Budán F. Probiotic Bacterium-Derived p40, p75, and HM0539 Proteins as Novel Postbiotics and Gut-Associated Immune System (GAIS) Modulation: Postbiotic-Gut-Health Axis. Microorganisms. (2024) 13:23. doi: 10.3390/microorganisms13010023 39858791 PMC11767761

[29] Medzhitov R. Recognition of microorganisms and activation of the immune response. Nature. (2007) 449:819–26. doi: 10.1038/nature06246 17943118

[30] Zhang H Wang M Zhao X Wang Y Chen X Su J. Role of stress in skin diseases: A neuroendocrine-immune interaction view. Brain Behav Immun. (2024) 116:286–302. doi: 10.1016/j.bbi.2023.12.005 38128623

[31] Ayasse MT Buddenkotte J Alam M Steinhoff M. Role of neuroimmune circuits and pruritus in psoriasis. Exp Dermatol. (2020) 29:414–26. doi: 10.1111/exd.14071 31954075

[32] Maruani A Samimi M Stembridge N Abdel Hay R Tavernier E Hughes C. Non-antistreptococcal interventions for acute guttate psoriasis or an acute guttate flare of chronic psoriasis. Cochrane Database Syst Rev. (2019) 4:Cd011541. doi: 10.1002/14651858.CD011541.pub2 30958563 PMC6452774

[33] Huang YW Tsai TF. HLA-cw1 and psoriasis. Am J Clin Dermatol. (2021) 22:339–47. doi: 10.1007/s40257-020-00585-1 PMC7812566 33460021

[34] De-Jesús-Gil C Ruiz-Romeu E Ferran M Chiriac A Deza G Hóllo P. CLA(+) T cell response to microbes in psoriasis. Front Immunol. (2018) 9:1488. doi: 10.3389/fimmu.2018.01488 30013558 PMC6036263

[35] Yan KX Fang X Han L Zhang ZH Kang KF Zheng ZZ. Foxp3+ regulatory T cells and related cytokines differentially expressed in plaque vs. guttate psoriasis vulgaris. Br J Dermatol. (2010) 163:48–56. doi: 10.1111/j.1365-2133.2010.09742.x 20222932

[36] Yan K Han L Deng H Fang X Zhang Z Huang G. The distinct role and regulatory mechanism of IL-17 and IFN-γ in the initiation and development of plaque vs guttate psoriasis. J Dermatol Sci. (2018) 92:106–13. doi: 10.1016/j.jdermsci.2018.07.001 30072243

[37] De-Jesús-Gil C Sans-de San Nicolás L Ruiz-Romeu E Ferran M Soria-Martinez L Chiriac A. Specific igA and CLA(+) T-cell IL-17 response to streptococcus pyogenes in psoriasis. J Invest Dermatol. (2020) 140:1364–1370.e1361. doi: 10.1016/j.jid.2019.12.022 31972247

[38] Ruiz-Romeu E Ferran M Sagristà M Gómez J Giménez-Arnau A Herszenyi K. Streptococcus pyogenes-induced cutaneous lymphocyte antigen-positive T cell-dependent epidermal cell activation triggers TH17 responses in patients with guttate psoriasis. J Allergy Clin Immunol. (2016) 138:491–499.e496. doi: 10.1016/j.jaci.2016.02.008 27056267

[39] Ruiz-Romeu E Ferran M de Jesús-Gil C García P Sagristà M Casanova JM. Microbe-dependent induction of IL-9 by CLA(+) T cells in psoriasis and relationship with IL-17A. J Invest Dermatol. (2018) 138:580–7. doi: 10.1016/j.jid.2017.08.048 29054600

[40] Armstrong AW Elston CA Elewski BE Ferris LK Gottlieb AB Lebwohl MG. Generalized pustular psoriasis: A consensus statement from the National Psoriasis Foundation. J Am Acad Dermatol. (2024) 90:727–30. doi: 10.1016/j.jaad.2023.09.080 37838256

[41] Prinz JC Choon SE Griffiths CEM Merola JF Morita A Ashcroft DM. Prevalence, comorbidities and mortality of generalized pustular psoriasis: A literature review. J Eur Acad Dermatol Venereol. (2023) 37:256–73. doi: 10.1111/jdv.18720 36331364

[42] Uppala R Tsoi LC Harms PW Wang B Billi AC Maverakis E. Autoinflammatory psoriasis”-genetics and biology of pustular psoriasis. Cell Mol Immunol. (2021) 18:307–17. doi: 10.1038/s41423-020-0519-3 PMC8027616 32814870

[43] Marrakchi S Puig L. Pathophysiology of generalized pustular psoriasis. Am J Clin Dermatol. (2022) 23:13–9. doi: 10.1007/s40257-021-00655-y PMC8801405 35061228

[44] Mansouri B Richards L Menter A. Treatment of two patients with generalized pustular psoriasis with the interleukin-1β inhibitor gevokizumab. Br J Dermatol. (2015) 173:239–41. doi: 10.1111/bjd.13614 25495649

[45] Armstrong AW Gooderham M Warren RB Papp KA Strober B Thaçi D. Treatment of plaque psoriasis with deucravacitinib (POETYK PSO-1 study): a plain language summary. Immunotherapy. (2023) 15:963–73. doi: 10.2217/imt-2023-0061 37150952

[46] Korman NJ. Management of psoriasis as a systemic disease: what is the evidence? Br J Dermatol. (2020) 182:840–8. doi: 10.1111/bjd.18245 PMC7187293 31225638

[47] Krueger JG Wharton KA Jr Schlitt T Suprun M Torene RI Jiang X. IL-17A inhibition by secukinumab induces early clinical, histopathologic, and molecular resolution of psoriasis. J Allergy Clin Immunol. (2019) 144:750–63. doi: 10.1016/j.jaci.2019.04.029 31129129

[48] Benezeder T Wolf P. Resolution of plaque-type psoriasis: what is left behind (and reinitiates the disease). Semin Immunopathol. (2019) 41:633–44. doi: 10.1007/s00281-019-00766-z PMC6881414 31673756

[49] Salimi S Yamauchi PS Thakur R Weinberg JM Kircik L Abdelmaksoud A. Interleukin 23p19 inhibitors in chronic plaque psoriasis with focus on mirikizumab: A narrative review. Dermatol Ther. (2020) 33:e13800. doi: 10.1111/dth.13800 32530083

[50] Gargiulo L Ibba L Malagoli P Balato A Bardazzi F Burlando M. Drug survival of IL-12/23, IL-17 and IL-23 inhibitors for moderate-to-severe plaque psoriasis: a retrospective multicenter real-world experience on 5932 treatment courses - IL PSO (Italian landscape psoriasis). Front Immunol. (2023) 14:1341708. doi: 10.3389/fimmu.2023.1341708 38274801 PMC10808601

[51] Song B Wu C Liu W Wang Y Ning X Guo L. Integrative serum proteomics analysis reveals distinct immune and cardiovascular profile dysregulation in erythrodermic psoriasis. Br J Dermatol. (2023) 189:769–71. doi: 10.1093/bjd/ljad283 37584181

[52] Shao S Wang G Maverakis E Gudjonsson JE. Targeted treatment for erythrodermic psoriasis: rationale and recent advances. Drugs. (2020) 80:525–34. doi: 10.1007/s40265-020-01283-2 PMC7167352 32180204

[53] Hoy SM. Deucravacitinib: first approval. Drugs. (2022) 82:1671–9. doi: 10.1007/s40265-022-01796-y PMC9676857 36401743

[54] Scher JU Ogdie A Merola JF Ritchlin C. Preventing psoriatic arthritis: focusing on patients with psoriasis at increased risk of transition. Nat Rev Rheumatol. (2019) 15:153–66. doi: 10.1038/s41584-019-0175-0 30742092

[55] Veale DJ Fearon U. The pathogenesis of psoriatic arthritis. Lancet. (2018) 391:2273–84. doi: 10.1016/s0140-6736(18)30830-4 29893226

[56] Poddubnyy D Jadon DR Van den Bosch F Mease PJ Gladman DD. Axial involvement in psoriatic arthritis: An update for rheumatologists. Semin Arthritis Rheum. (2021) 51:880–7. doi: 10.1016/j.semarthrit.2021.06.006 34198146

[57] Gottlieb AB Merola JF. Axial psoriatic arthritis: An update for dermatologists. J Am Acad Dermatol. (2021) 84:92–101. doi: 10.1016/j.jaad.2020.05.089 32747079

[58] Kopp T Riedl E Bangert C Bowman EP Greisenegger E Horowitz A. Clinical improvement in psoriasis with specific targeting of interleukin-23. Nature. (2015) 521:222–6. doi: 10.1038/nature14175 25754330

[59] Tait Wojno ED Hunter CA Stumhofer JS. The immunobiology of the interleukin-12 family: room for discovery. Immunity. (2019) 50:851–70. doi: 10.1016/j.immuni.2019.03.011 PMC6472917 30995503

[60] Teng MW Bowman EP McElwee JJ Smyth MJ Casanova JL Cooper AM. IL-12 and IL-23 cytokines: from discovery to targeted therapies for immune-mediated inflammatory diseases. Nat Med. (2015) 21:719–29. doi: 10.1038/nm.3895 26121196

[61] Chyuan IT Lai JH. New insights into the IL-12 and IL-23: From a molecular basis to clinical application in immune-mediated inflammation and cancers. Biochem Pharmacol. (2020) 175:113928. doi: 10.1016/j.bcp.2020.113928 32217101

[62] Lee E Trepicchio WL Oestreicher JL Pittman D Wang F Chamian F. Increased expression of interleukin 23 p19 and p40 in lesional skin of patients with psoriasis vulgaris. J Exp Med. (2004) 199:125–30. doi: 10.1084/jem.20030451 PMC1887731 14707118

[63] Lowes MA Suarez-Farinas M Krueger JG. Immunology of psoriasis. Annu Rev Immunol. (2014) 32:227–55. doi: 10.1146/annurev-immunol-032713-120225 PMC4229247 24655295

[64] Diani M Altomare G Reali E. T cell responses in psoriasis and psoriatic arthritis. Autoimmun Rev. (2015) 14:286–92. doi: 10.1016/j.autrev.2014.11.012 25445403

[65] Yang J Sundrud MS Skepner J Yamagata T. Targeting Th17 cells in autoimmune diseases. Trends Pharmacol Sci. (2014) 35:493–500. doi: 10.1016/j.tips.2014.07.006 25131183

[66] Miossec P Kolls JK. Targeting IL-17 and TH17 cells in chronic inflammation. Nat Rev Drug Discovery. (2012) 11:763–76. doi: 10.1038/nrd3794 23023676

[67] Hu P Wang M Gao H Zheng A Li J Mu D. The role of helper T cells in psoriasis. Front Immunol. (2021) 12:788940. doi: 10.3389/fimmu.2021.788940 34975883 PMC8714744

[68] Lowes MA Russell CB Martin DA Towne JE Krueger JG. The IL-23/T17 pathogenic axis in psoriasis is amplified by keratinocyte responses. Trends Immunol. (2013) 34:174–81. doi: 10.1016/j.it.2012.11.005 PMC3721313 23291100

[69] Hawkes JE Yan BY Chan TC Krueger JG. Discovery of the IL-23/IL-17 signaling pathway and the treatment of psoriasis. J Immunol. (2018) 201:1605–13. doi: 10.4049/jimmunol.1800013 PMC6129988 30181299

[70] Singh TP Zhang HH Borek I Wolf P Hedrick MN Singh SP. Monocyte-derived inflammatory Langerhans cells and dermal dendritic cells mediate psoriasis-like inflammation. Nat Commun. (2016) 7:13581. doi: 10.1038/ncomms13581 27982014 PMC5171657

[71] Wang Y Edelmayer R Wetter J Salte K Gauvin D Leys L. Monocytes/Macrophages play a pathogenic role in IL-23 mediated psoriasis-like skin inflammation. Sci Rep. (2019) 9:5310. doi: 10.1038/s41598-019-41655-7 30926837 PMC6441056

[72] Detmar M Brown LF Claffey KP Yeo KT Kocher O Jackman RW. Overexpression of vascular permeability factor/vascular endothelial growth factor and its receptors in psoriasis. J Exp Med. (1994) 180:1141–6. doi: 10.1084/jem.180.3.1141 PMC2191647 8064230

[73] Schonthaler HB Huggenberger R Wculek SK Detmar M Wagner EF. Systemic anti-VEGF treatment strongly reduces skin inflammation in a mouse model of psoriasis. Proc Natl Acad Sci U.S.A. (2009) 106:21264–9. doi: 10.1073/pnas.0907550106 PMC2795522 19995970

[74] Xia YP Li B Hylton D Detmar M Yancopoulos GD Rudge JS. Transgenic delivery of VEGF to mouse skin leads to an inflammatory condition resembling human psoriasis. Blood. (2003) 102:161–8. doi: 10.1182/blood-2002-12-3793 12649136

[75] Canavese M Altruda F Ruzicka T Schauber J. Vascular endothelial growth factor (VEGF) in the pathogenesis of psoriasis–a possible target for novel therapies? J Dermatol Sci. (2010) 58:171–6. doi: 10.1016/j.jdermsci.2010.03.023 20430590

[76] Gupta RK Gracias DT Figueroa DS Miki H Miller J Fung K. TWEAK functions with TNF and IL-17 on keratinocytes and is a potential target for psoriasis therapy. Sci Immunol. (2021) 6:eabi8823. doi: 10.1126/sciimmunol.abi8823 34797693 PMC8756771

[77] Zhou L Wang Y Wan Q Wu F Barbon J Dunstan R. A non-clinical comparative study of IL-23 antibodies in psoriasis. MAbs. (2021) 13:1964420. doi: 10.1080/19420862.2021.1964420 34460338 PMC8409790

[78] Brembilla NC Senra L Boehncke WH. The IL-17 family of cytokines in psoriasis: IL-17A and beyond. Front Immunol. (2018) 9:1682. doi: 10.3389/fimmu.2018.01682 30127781 PMC6088173

[79] Blauvelt A Chiricozzi A. The immunologic role of IL-17 in psoriasis and psoriatic arthritis pathogenesis. Clin Rev Allergy Immunol. (2018) 55:379–90. doi: 10.1007/s12016-018-8702-3 PMC6244934 30109481

[80] Matos TR O'Malley JT Lowry EL Hamm D Kirsch IR Robins HS. Clinically resolved psoriatic lesions contain psoriasis-specific IL-17-producing alphabeta T cell clones. J Clin Invest. (2017) 127:4031–41. doi: 10.1172/JCI93396 PMC5663366 28945199

[81] Monin L Gaffen SL. Interleukin 17 family cytokines: signaling mechanisms, biological activities, and therapeutic implications. Cold Spring Harb Perspect Biol. (2018) 10:a028522. doi: 10.1101/cshperspect.a028522 28620097 PMC5732092

[82] Raychaudhuri SP. Role of IL-17 in psoriasis and psoriatic arthritis. Clin Rev Allergy Immunol. (2013) 44:183–93. doi: 10.1007/s12016-012-8307-1 22362575

[83] Canavan TN Elmets CA Cantrell WL Evans JM Elewski BE. Anti-IL-17 medications used in the treatment of plaque psoriasis and psoriatic arthritis: A comprehensive review. Am J Clin Dermatol. (2016) 17:33–47. doi: 10.1007/s40257-015-0162-4 26649440

[84] Hawkes JE Chan TC Krueger JG. Psoriasis pathogenesis and the development of novel targeted immune therapies. J Allergy Clin Immunol. (2017) 140:645–53. doi: 10.1016/j.jaci.2017.07.004 PMC5600287 28887948

[85] Nestle FO Kaplan DH Barker J. Psoriasis. N Engl J Med. (2009) 361:496–509. doi: 10.1056/NEJMra0804595 19641206

[86] Moos S Mohebiany AN Waisman A Kurschus FC. Imiquimod-induced psoriasis in mice depends on the IL-17 signaling of keratinocytes. J Invest Dermatol. (2019) 139:1110–7. doi: 10.1016/j.jid.2019.01.006 30684554

[87] Harper EG Guo C Rizzo H Lillis JV Kurtz SE Skorcheva I. Th17 cytokines stimulate CCL20 expression in keratinocytes *in vitro* and *in vivo*: implications for psoriasis pathogenesis. J Invest Dermatol. (2009) 129:2175–83. doi: 10.1038/jid.2009.65 PMC2892172 19295614

[88] Furue M Furue K Tsuji G Nakahara T. Interleukin-17A and keratinocytes in psoriasis. Int J Mol Sci. (2020) 21:1275. doi: 10.3390/ijms21041275 32070069 PMC7072868

[89] Ekman AK Bivik Eding C Rundquist I Enerback C. IL-17 and IL-22 promote keratinocyte stemness in the germinative compartment in psoriasis. J Invest Dermatol. (2019) 139:1564–1573 e1568. doi: 10.1016/j.jid.2019.01.014 30684548

[90] Mahil SK Catapano M Di Meglio P Dand N Ahlfors H Carr IM. An analysis of IL-36 signature genes and individuals with IL1RL2 knockout mutations validates IL-36 as a psoriasis therapeutic target. Sci Transl Med. (2017) 9:eaan2514. doi: 10.1126/scitranslmed.aan2514 29021166

[91] Johnston A Fritz Y Dawes SM Diaconu D Al-Attar PM Guzman AM. Keratinocyte overexpression of IL-17C promotes psoriasiform skin inflammation. J Immunol. (2013) 190:2252–62. doi: 10.4049/jimmunol.1201505 PMC3577967 23359500

[92] Bridgewood C Fearnley GW Berekmeri A Laws P Macleod T Ponnambalam S. IL-36gamma is a strong inducer of IL-23 in psoriatic cells and activates angiogenesis. Front Immunol. (2018) 9:200. doi: 10.3389/fimmu.2018.00200 29535706 PMC5834930

[93] Blumberg H Conklin D Xu WF Grossmann A Brender T Carollo S. Interleukin 20: discovery, receptor identification, and role in epidermal function. Cell. (2001) 104:9–19. doi: 10.1016/s0092-8674(01)00187-8 11163236

[94] Wolk K Haugen HS Xu W Witte E Waggie K Anderson M. IL-22 and IL-20 are key mediators of the epidermal alterations in psoriasis while IL-17 and IFN-gamma are not. J Mol Med (Berl). (2009) 87:523–36. doi: 10.1007/s00109-009-0457-0 19330474

[95] Sabat R Wolk K. Research in practice: IL-22 and IL-20: significance for epithelial homeostasis and psoriasis pathogenesis. J Dtsch Dermatol Ges. (2011) 9:518–23. doi: 10.1111/j.1610-0387.2011.07611.x 21251229

[96] Witte E Kokolakis G Witte K Philipp S Doecke WD Babel N. IL-19 is a component of the pathogenetic IL-23/IL-17 cascade in psoriasis. J Invest Dermatol. (2014) 134:2757–67. doi: 10.1038/jid.2014.308 25046339

[97] Xu X Prens E Florencia E Leenen P Boon L Asmawidjaja P. Interleukin-17A drives IL-19 and IL-24 expression in skin stromal cells regulating keratinocyte proliferation. Front Immunol. (2021) 12:719562. doi: 10.3389/fimmu.2021.719562 34616394 PMC8488340

[98] Gaffen SL. Structure and signalling in the IL-17 receptor family. Nat Rev Immunol. (2009) 9:556–67. doi: 10.1038/nri2586 PMC2821718 19575028

[99] McGeachy MJ Cua DJ Gaffen SL. The IL-17 family of cytokines in health and disease. Immunity. (2019) 50:892–906. doi: 10.1016/j.immuni.2019.03.021 30995505 PMC6474359

[100] Tseng JC Chang YC Huang CM Hsu LC Chuang TH. Therapeutic development based on the immunopathogenic mechanisms of psoriasis. Pharmaceutics. (2021) 13:1064. doi: 10.3390/pharmaceutics13071064 34371756 PMC8308930

[101] Johansen C Mose M Ommen P Bertelsen T Vinter H Hailfinger S. IkappaBzeta is a key driver in the development of psoriasis. Proc Natl Acad Sci U.S.A. (2015) 112:E5825–5833. doi: 10.1073/pnas.1509971112 PMC4629387 26460049

[102] Sabat R Wolk K Loyal L Docke WD Ghoreschi K. T cell pathology in skin inflammation. Semin Immunopathol. (2019) 41:359–77. doi: 10.1007/s00281-019-00742-7 PMC6505509 31028434

[103] Sieminska I Pieniawska M Grzywa TM. The immunology of psoriasis-current concepts in pathogenesis. Clin Rev Allergy Immunol. (2024) 66:164–91. doi: 10.1007/s12016-024-08991-7 PMC11193704 38642273

[104] Suzuki E Mellins ED Gershwin ME Nestle FO Adamopoulos IE. The IL-23/IL-17 axis in psoriatic arthritis. Autoimmun Rev. (2014) 13:496–502. doi: 10.1016/j.autrev.2014.01.050 24424175 PMC3995976

[105] Dyring-Andersen B Honoré TV Madelung A Bzorek M Simonsen S Clemmensen SN. Interleukin (IL)-17A and IL-22-producing neutrophils in psoriatic skin. Br J Dermatol. (2017) 177:e321–2. doi: 10.1111/bjd.15533 PMC5921865 28369663

[106] Cavagnero KJ Li F Dokoshi T Nakatsuji T O'Neill AM Aguilera C. CXCL12+ dermal fibroblasts promote neutrophil recruitment and host defense by recognition of IL-17. J Exp Med. (2024) 221:e20231425. doi: 10.1084/jem.20231425 38393304 PMC10890925

[107] Boehncke WH Schon MP. Psoriasis. Lancet. (2015) 386:983–94. doi: 10.1016/S0140-6736(14)61909-7 26025581

[108] Wang X Kaiser H Kvist-Hansen A McCauley BD Skov L Hansen PR. IL-17 pathway members as potential biomarkers of effective systemic treatment and cardiovascular disease in patients with moderate-to-severe psoriasis. Int J Mol Sci. (2022) 23:555. doi: 10.3390/ijms23010555 35008981 PMC8745093

[109] Tollenaere MAX Hebsgaard J Ewald DA Lovato P Garcet S Li X. Signalling of multiple interleukin (IL)-17 family cytokines via IL-17 receptor A drives psoriasis-related inflammatory pathways. Br J Dermatol. (2021) 185:585–94. doi: 10.1111/bjd.20090 PMC8453543 33792895

[110] Rose-John S Jenkins BJ Garbers C Moll JM Scheller J. Targeting IL-6 trans-signalling: past, present and future prospects. Nat Rev Immunol. (2023) 23:666–81. doi: 10.1038/s41577-023-00856-y PMC10108826 37069261

[111] Mihara M Hashizume M Yoshida H Suzuki M Shiina M. IL-6/IL-6 receptor system and its role in physiological and pathological conditions. Clin Sci (Lond). (2012) 122:143–59. doi: 10.1042/CS20110340 22029668

[112] Kang S Narazaki M Metwally H Kishimoto T. Historical overview of the interleukin-6 family cytokine. J Exp Med. (2020) 217:e20190347. doi: 10.1084/jem.20190347 32267936 PMC7201933

[113] Blauvelt A. IL-6 differs from TNF-alpha: unpredicted clinical effects caused by IL-6 blockade in psoriasis. J Invest Dermatol. (2017) 137:541–2. doi: 10.1016/j.jid.2016.11.022 28235443

[114] Chen Y Miao X Xiang Y Kuai L Ding X Ma T. Qinzhu Liangxue inhibits IL-6-induced hyperproliferation and inflammation in HaCaT cells by regulating METTL14/SOCS3/STAT3 axis. J Ethnopharmacol. (2023) 317:116809. doi: 10.1016/j.jep.2023.116809 37336334

[115] Maddur MS Miossec P Kaveri SV Bayry J. Th17 cells: biology, pathogenesis of autoimmune and inflammatory diseases, and therapeutic strategies. Am J Pathol. (2012) 181:8–18. doi: 10.1016/j.ajpath.2012.03.044 22640807

[116] Barygina V Becatti M Prignano F Lotti T Taddei N Fiorillo C. Fibroblasts to keratinocytes redox signaling: the possible role of ROS in psoriatic plaque formation. Antioxid (Basel). (2019) 8:566. doi: 10.3390/antiox8110566 PMC6912201 31752190

[117] Yang L Anderson DE Baecher-Allan C Hastings WD Bettelli E Oukka M. IL-21 and TGF-beta are required for differentiation of human T(H)17 cells. Nature. (2008) 454:350–2. doi: 10.1038/nature07021 PMC2760130 18469800

[118] Veldhoen M Hocking RJ Atkins CJ Locksley RM Stockinger B. TGFbeta in the context of an inflammatory cytokine milieu supports *de novo* differentiation of IL-17-producing T cells. Immunity. (2006) 24:179–89. doi: 10.1016/j.immuni.2006.01.001 16473830

[119] McGeachy MJ Chen Y Tato CM Laurence A Joyce-Shaikh B Blumenschein WM. The interleukin 23 receptor is essential for the terminal differentiation of interleukin 17-producing effector T helper cells in *vivo* . Nat Immunol. (2009) 10:314–24. doi: 10.1038/ni.1698 PMC2945605 19182808

[120] Veldhoen M Hocking RJ Flavell RA Stockinger B. Signals mediated by transforming growth factor-beta initiate autoimmune encephalomyelitis, but chronic inflammation is needed to sustain disease. Nat Immunol. (2006) 7:1151–6. doi: 10.1038/ni1391 16998492

[121] Goldstein JD Bassoy EY Caruso A Palomo J Rodriguez E Lemeille S. IL-36 signaling in keratinocytes controls early IL-23 production in psoriasis-like dermatitis. Life Sci Alliance. (2020) 3:e202000688. doi: 10.26508/lsa.202000688 32345660 PMC7190273

[122] Ma F Plazyo O Billi AC Tsoi LC Xing X Wasikowski R. Single cell and spatial sequencing define processes by which keratinocytes and fibroblasts amplify inflammatory responses in psoriasis. Nat Commun. (2023) 14:3455. doi: 10.1038/s41467-023-39020-4 37308489 PMC10261041

[123] Madonna S Girolomoni G Dinarello CA Albanesi C. The significance of IL-36 hyperactivation and IL-36R targeting in psoriasis. Int J Mol Sci. (2019) 20:3318. doi: 10.3390/ijms20133318 31284527 PMC6650959

[124] Iznardo H Puig L. Exploring the role of IL-36 cytokines as a new target in psoriatic disease. Int J Mol Sci. (2021) 22:4344. doi: 10.3390/ijms22094344 33919434 PMC8122427

[125] Hojen JF Kristensen MLV McKee AS Wade MT Azam T Lunding LP. IL-1R3 blockade broadly attenuates the functions of six members of the IL-1 family, revealing their contribution to models of disease. Nat Immunol. (2019) 20:1138–49. doi: 10.1038/s41590-019-0467-1 PMC6707854 31427775

[126] Hu X Qi C Feng F Wang Y Di T Meng Y. Combining network pharmacology, RNA-seq, and metabolomics strategies to reveal the mechanism of Cimicifugae Rhizoma - Smilax glabra Roxb herb pair for the treatment of psoriasis. Phytomedicine. (2022) 105:154384. doi: 10.1016/j.phymed.2022.154384 35963195

[127] Frey S Derer A Messbacher ME Baeten DL Bugatti S Montecucco C. The novel cytokine interleukin-36alpha is expressed in psoriatic and rheumatoid arthritis synovium. Ann Rheum Dis. (2013) 72:1569–74. doi: 10.1136/annrheumdis-2012-202264 23268368

[128] Wolk K Brembach TC Simaite D Bartnik E Cucinotta S Pokrywka A. Activity and components of the granulocyte colony-stimulating factor pathway in hidradenitis suppurativa. Br J Dermatol. (2021) 185:164–76. doi: 10.1111/bjd.19795 33400270

[129] Elewski BE Lebwohl MG Anadkat MJ Barker J Ghoreschi K Imafuku S. Rapid and sustained improvements in Generalized Pustular Psoriasis Physician Global Assessment scores with spesolimab for treatment of generalized pustular psoriasis flares in the randomized, placebo-controlled Effisayil 1 study. J Am Acad Dermatol. (2023) 89:36–44. doi: 10.1016/j.jaad.2023.02.040 36870370

[130] Arakawa A Vollmer S Besgen P Galinski A Summer B Kawakami Y. Unopposed IL-36 activity promotes clonal CD4(+) T-cell responses with IL-17A production in generalized pustular psoriasis. J Invest Dermatol. (2018) 138:1338–47. doi: 10.1016/j.jid.2017.12.024 29288651

[131] Akiyama M. Pustular psoriasis as an autoinflammatory keratinization disease (AiKD): Genetic predisposing factors and promising therapeutic targets. J Dermatol Sci. (2022) 105:11–7. doi: 10.1016/j.jdermsci.2021.11.009 34973880

[132] Ni X Xu Y Wang W Kong B Ouyang J Chen J. IL-17D-induced inhibition of DDX5 expression in keratinocytes amplifies IL-36R-mediated skin inflammation. Nat Immunol. (2022) 23:1577–87. doi: 10.1038/s41590-022-01339-3 PMC9663298 36271146

[133] Moseley TA Haudenschild DR Rose L Reddi AH. Interleukin-17 family and IL-17 receptors. Cytokine Growth Factor Rev. (2003) 14:155–74. doi: 10.1016/s1359-6101(03)00002-9 12651226

[134] Hovhannisyan Z Liu N Khalil-Aguero S Panea C VanValkenburgh J Zhang R. Enhanced IL-36R signaling promotes barrier impairment and inflammation in skin and intestine. Sci Immunol. (2020) 5:eaax1686. doi: 10.1126/sciimmunol.aax1686 33443029

[135] Han MM Yuan XR Shi X Zhu XY Su Y Xiong DK. The pathological mechanism and potential application of IL-38 in autoimmune diseases. Front Pharmacol. (2021) 12:732790. doi: 10.3389/fphar.2021.732790 34539413 PMC8443783

[136] Boutet MA Nerviani A Pitzalis C. IL-36, IL-37, and IL-38 cytokines in skin and joint inflammation: A comprehensive review of their therapeutic potential. Int J Mol Sci. (2019) 20:1257. doi: 10.3390/ijms20061257 30871134 PMC6470667

[137] Puig L Costanzo A Munoz-Elias EJ Jazra M Wegner S Paul CF. The biological basis of disease recurrence in psoriasis: a historical perspective and current models. Br J Dermatol. (2022) 186:773–81. doi: 10.1111/bjd.20963 PMC9374062 34939663

[138] Garraud T Harel M Boutet MA Le Goff B Blanchard F. The enigmatic role of IL-38 in inflammatory diseases. Cytokine Growth Factor Rev. (2018) 39:26–35. doi: 10.1016/j.cytogfr.2018.01.001 29366546

[139] Perumal NB Kaplan MH. Regulating Il9 transcription in T helper cells. Trends Immunol. (2011) 32:146–50. doi: 10.1016/j.it.2011.01.006 PMC3070825 21371941

[140] Midde HS Priyadarssini M Rajappa M Munisamy M Raj Mohan PS Singh S. Interleukin-9 serves as a key link between systemic inflammation and angiogenesis in psoriasis. Clin Exp Dermatol. (2021) 46:50–7. doi: 10.1111/ced.14335 32516443

[141] Ciccia F Guggino G Ferrante A Raimondo S Bignone R Rodolico V. Interleukin-9 overexpression and th9 polarization characterize the inflamed gut, the synovial tissue, and the peripheral blood of patients with psoriatic arthritis. Arthritis Rheumatol. (2016) 68:1922–31. doi: 10.1002/art.39649 26895441

[142] Angkasekwinai P Dong C. IL-9-producing T cells: potential players in allergy and cancer. Nat Rev Immunol. (2021) 21:37–48. doi: 10.1038/s41577-020-0396-0 32788707

[143] Kaplan MH Hufford MM Olson MR. The development and *in vivo* function of T helper 9 cells. Nat Rev Immunol. (2015) 15:295–307. doi: 10.1038/nri3824 25848755 PMC4445728

[144] Kamiya S Ikegami I Yanagi M Takaki H Kamekura R Sato T. Functional interplay between IL-9 and peptide YY contributes to chronic skin inflammation. J Invest Dermatol. (2022) 142:3222–3231 e3225. doi: 10.1016/j.jid.2022.06.021 35850207

[145] Feng B Pan B Huang J Du Y Wang X Wu J. PDE4D/cAMP/IL-23 axis determines the immunotherapy efficacy of lung adenocarcinoma via activating the IL-9 autocrine loop of cytotoxic T lymphocytes. Cancer Lett. (2023) 565:216224. doi: 10.1016/j.canlet.2023.216224 37196909

[146] Trinchieri G. Interleukin-12 and the regulation of innate resistance and adaptive immunity. Nat Rev Immunol. (2003) 3:133–46. doi: 10.1038/nri1001 12563297

[147] Jin J Xie X Xiao Y Hu H Zou Q Cheng X. Epigenetic regulation of the expression of Il12 and Il23 and autoimmune inflammation by the deubiquitinase Trabid. Nat Immunol. (2016) 17:259–68. doi: 10.1038/ni.3347 PMC4755875 26808229

[148] Parham C Chirica M Timans J Vaisberg E Travis M Cheung J. A receptor for the heterodimeric cytokine IL-23 is composed of IL-12Rbeta1 and a novel cytokine receptor subunit, IL-23R. J Immunol. (2002) 168:5699–708. doi: 10.4049/jimmunol.168.11.5699 12023369

[149] Watford WT Hissong BD Bream JH Kanno Y Muul L O'Shea JJ. Signaling by IL-12 and IL-23 and the immunoregulatory roles of STAT4. Immunol Rev. (2004) 202:139–56. doi: 10.1111/j.0105-2896.2004.00211.x 15546391

[150] Cibrian D Saiz ML Fuente H Sanchez-Diaz R Moreno-Gonzalo O Jorge I. Erratum: CD69 controls the uptake of L-tryptophan through LAT1-CD98 and AhR-dependent secretion of IL-22 in psoriasis. Nat Immunol. (2016) 17:1235. doi: 10.1038/ni1016-1235c 27648548

[151] Nograles KE Zaba LC Guttman-Yassky E Fuentes-Duculan J Suarez-Farinas M Cardinale I. Th17 cytokines interleukin (IL)-17 and IL-22 modulate distinct inflammatory and keratinocyte-response pathways. Br J Dermatol. (2008) 159:1092–102. doi: 10.1111/j.1365-2133.2008.08769.x PMC2724264 18684158

[152] Rutz S Noubade R Eidenschenk C Ota N Zeng W Zheng Y. Transcription factor c-Maf mediates the TGF-beta-dependent suppression of IL-22 production in T(H)17 cells. Nat Immunol. (2011) 12:1238–45. doi: 10.1038/ni.2134 22001828

[153] Kotenko SV Izotova LS Mirochnitchenko OV Esterova E Dickensheets H Donnelly RP. Identification of the functional interleukin-22 (IL-22) receptor complex: the IL-10R2 chain (IL-10Rbeta) is a common chain of both the IL-10 and IL-22 (IL-10-related T cell-derived inducible factor, IL-TIF) receptor complexes. J Biol Chem. (2001) 276:2725–32. doi: 10.1074/jbc.M007837200 11035029

[154] Boniface K Bernard FX Garcia M Gurney AL Lecron JC Morel F. IL-22 inhibits epidermal differentiation and induces proinflammatory gene expression and migration of human keratinocytes. J Immunol. (2005) 174:3695–702. doi: 10.4049/jimmunol.174.6.3695 15749908

[155] Sa SM Valdez PA Wu J Jung K Zhong F Hall L. The effects of IL-20 subfamily cytokines on reconstituted human epidermis suggest potential roles in cutaneous innate defense and pathogenic adaptive immunity in psoriasis. J Immunol. (2007) 178:2229–40. doi: 10.4049/jimmunol.178.4.2229 17277128

[156] Dumoutier L Van Roost E Colau D Renauld JC. Human interleukin-10-related T cell-derived inducible factor: molecular cloning and functional characterization as an hepatocyte-stimulating factor. Proc Natl Acad Sci U.S.A. (2000) 97:10144–9. doi: 10.1073/pnas.170291697 PMC27764 10954742

[157] Romer J Hasselager E Norby PL Steiniche T Thorn Clausen J Kragballe K. Epidermal overexpression of interleukin-19 and -20 mRNA in psoriatic skin disappears after short-term treatment with cyclosporine a or calcipotriol. J Invest Dermatol. (2003) 121:1306–11. doi: 10.1111/j.1523-1747.2003.12626.x 14675174

[158] Kunz S Wolk K Witte E Witte K Doecke WD Volk HD. Interleukin (IL)-19, IL-20 and IL-24 are produced by and act on keratinocytes and are distinct from classical ILs. Exp Dermatol. (2006) 15:991–1004. doi: 10.1111/j.1600-0625.2006.00516.x 17083366

[159] Sabat R Ouyang W Wolk K. Therapeutic opportunities of the IL-22-IL-22R1 system. Nat Rev Drug Discovery. (2014) 13:21–38. doi: 10.1038/nrd4176 24378801

[160] Beutler B Cerami A. The biology of cachectin/TNF–a primary mediator of the host response. Annu Rev Immunol. (1989) 7:625–55. doi: 10.1146/annurev.iy.07.040189.003205 2540776

[161] Medler J Wajant H. Tumor necrosis factor receptor-2 (TNFR2): an overview of an emerging drug target. Expert Opin Ther Targets. (2019) 23:295–307. doi: 10.1080/14728222.2019.1586886 30856027

[162] Siegmund D Wajant H. TNF and TNF receptors as therapeutic targets for rheumatic diseases and beyond. Nat Rev Rheumatol. (2023) 19:576–91. doi: 10.1038/s41584-023-01002-7 37542139

[163] Grell M Douni E Wajant H Lohden M Clauss M Maxeiner B. The transmembrane form of tumor necrosis factor is the prime activating ligand of the 80 kDa tumor necrosis factor receptor. Cell. (1995) 83:793–802. doi: 10.1016/0092-8674(95)90192-2 8521496

[164] Sheehan KC Pinckard JK Arthur CD Dehner LP Goeddel DV Schreiber RD. Monoclonal antibodies specific for murine p55 and p75 tumor necrosis factor receptors: identification of a novel *in vivo* role for p75. J Exp Med. (1995) 181:607–17. doi: 10.1084/jem.181.2.607 PMC2191879 7836916

[165] Li P Zheng Y Chen X. Drugs for autoimmune inflammatory diseases: from small molecule compounds to anti-TNF biologics. Front Pharmacol. (2017) 8:460. doi: 10.3389/fphar.2017.00460 28785220 PMC5506195

[166] Fischer R Kontermann RE Pfizenmaier K. Selective targeting of TNF receptors as a novel therapeutic approach. Front Cell Dev Biol. (2020) 8:401. doi: 10.3389/fcell.2020.00401 32528961 PMC7264106

[167] Leone GM Mangano K Petralia MC Nicoletti F Fagone P. Past, present and (Foreseeable) future of biological anti-TNF alpha therapy. J Clin Med. (2023) 12:1630. doi: 10.3390/jcm12041630 36836166 PMC9963154

[168] Ding H Wang G Yu Z Sun H Wang L. Role of interferon-gamma (IFN-γ) and IFN-γ receptor 1/2 (IFNγR1/2) in regulation of immunity, infection, and cancer development: IFN-γ-dependent or independent pathway. BioMed Pharmacother. (2022) 155:113683. doi: 10.1016/j.biopha.2022.113683 36095965

[169] Ivashkiv LB. IFNγ: signalling, epigenetics and roles in immunity, metabolism, disease and cancer immunotherapy. Nat Rev Immunol. (2018) 18:545–58. doi: 10.1038/s41577-018-0029-z PMC6340644 29921905

[170] Dai H Adamopoulos IE. Psoriatic arthritis under the influence of IFNγ. Clin Immunol. (2020) 218:108513. doi: 10.1016/j.clim.2020.108513 32574710 PMC7595649

[171] Della Bella C Corrà A Mantengoli E Galano A Benagiano M Bonciani D. Skin IL-17A and IFN-γ Production correlate with disease severity in patients with psoriasis and streptococcal infection. J Invest Dermatol. (2023) 143:925–32. doi: 10.1016/j.jid.2022.10.025 36642401

[172] Wang CQ Haxhinasto S Garcet S Kunjravia N Cueto I Gonzalez J. Comparison of the inflammatory circuits in psoriasis vulgaris, non–Pustular palmoplantar psoriasis, and palmoplantar pustular psoriasis. J Invest Dermatol. (2023) 143:87–97.e14. doi: 10.1016/j.jid.2022.05.1094 35934055

[173] Srivastava A Luo L Lohcharoenkal W Meisgen F Pasquali L Pivarcsi A. Cross-talk between IFN-γ and TWEAK through miR-149 amplifies skin inflammation in psoriasis. J Allergy Clin Immunol. (2021) 147:2225–35. doi: 10.1016/j.jaci.2020.12.657 33705829

[174] Luque-Martin R Angell DC Kalxdorf M Bernard S Thompson W Eberl HC. IFN-γ Drives human monocyte differentiation into highly proinflammatory macrophages that resemble a phenotype relevant to psoriasis. J Immunol. (2021) 207:555–68. doi: 10.4049/jimmunol.2001310 34233910

[175] Clement CC D'Alessandro A Thangaswamy S Chalmers S Furtado R Spada S. 3-hydroxy-L-kynurenamine is an immunomodulatory biogenic amine. Nat Commun. (2021) 12:4447. doi: 10.1038/s41467-021-24785-3 34290243 PMC8295276

[176] Li Y Cui H Li S Li X Guo H Nandakumar KS. Kaempferol modulates IFN-γ induced JAK-STAT signaling pathway and ameliorates imiquimod-induced psoriasis-like skin lesions. Int Immunopharmacol. (2023) 114:109585. doi: 10.1016/j.intimp.2022.109585 36527884

[177] Lv J Zhou D Wang Y Sun W Zhang C Xu J. Effects of luteolin on treatment of psoriasis by repressing HSP90. Int Immunopharmacol. (2020) 79:106070. doi: 10.1016/j.intimp.2019.106070 31918062

[178] Morikawa M Derynck R Miyazono K. TGF-β and the TGF-β Family: context-dependent roles in cell and tissue physiology. Cold Spring Harbor Perspect Biol. (2016) 8:a021873. doi: 10.1101/cshperspect.a021873 PMC4852809 27141051

[179] Machelak W Szczepaniak A Jacenik D Zielińska M. The role of GDF11 during inflammation – An overview. Life Sci. (2023) 322:121650. doi: 10.1016/j.lfs.2023.121650 37011872

[180] Cuesta-Gomez N Medina-Ruiz L Graham GJ Campbell JDM. IL-6 and TGF-β-secreting adoptively-transferred murine mesenchymal stromal cells accelerate healing of psoriasis-like skin inflammation and upregulate IL-17A and TGF-β. Int J Mol Sci. (2023) 24:10132. doi: 10.3390/ijms241210132 37373278 PMC10298958

[181] Yang L Zhang S Wang G. Keratin 17 in disease pathogenesis: from cancer to dermatoses. J Pathol. (2018) 247:158–65. doi: 10.1002/path.5178 30306595

[182] Zhang J He L Wang Z Shao S Qiao P Zhang J. Decreasing GDF15 promotes inflammatory signals and neutrophil infiltration in psoriasis models. J Invest Dermatol. (2023) 143:419–430.e418. doi: 10.1016/j.jid.2022.07.026 36049542

[183] He H Bissonnette R Wu J Diaz A Saint-Cyr Proulx E Maari C. Tape strips detect distinct immune and barrier profiles in atopic dermatitis and psoriasis. J Allergy Clin Immunol. (2021) 147:199–212. doi: 10.1016/j.jaci.2020.05.048 32709423

[184] Ramessur R Corbett M Marshall D Acencio ML Barbosa IA Dand N. Biomarkers of disease progression in people with psoriasis: a scoping review. Br J Dermatol. (2022) 187:481–93. doi: 10.1111/bjd.21627 PMC9796834 35482474

[185] Karin N. CXCR3 ligands in cancer and autoimmunity, chemoattraction of effector T cells, and beyond. Front Immunol. (2020) 11:976. doi: 10.3389/fimmu.2020.00976 32547545 PMC7274023

[186] Antonelli A Ferrari SM Giuggioli D Ferrannini E Ferri C Fallahi P. Chemokine (C–X–C motif) ligand (CXCL)10 in autoimmune diseases. Autoimmun Rev. (2014) 13:272–80. doi: 10.1016/j.autrev.2013.10.010 24189283

[187] Tokunaga R Zhang W Naseem M Puccini A Berger MD Soni S. CXCL9, CXCL10, CXCL11/CXCR3 axis for immune activation - A target for novel cancer therapy. Cancer Treat Rev. (2018) 63:40–7. doi: 10.1016/j.ctrv.2017.11.007 PMC5801162 29207310

[188] Wu H Zeng L Ou J Wang T Chen Y Nandakumar KS. Estrogen acts through estrogen receptor-β to promote mannan-induced psoriasis-like skin inflammation. Front Immunol. (2022) 13:818173. doi: 10.3389/fimmu.2022.818173 35663991 PMC9160234

[189] Lebwohl M Ting PT Koo JY. Psoriasis treatment: traditional therapy. Ann Rheum Dis. (2005) 64 Suppl 2:ii83–86. doi: 10.1136/ard.2004.030791 PMC1766882 15708945

[190] Alam MS Gaida MM Ogawa Y Kolios AG Lasitschka F Ashwell JD. Counter-regulation of T cell effector function by differentially activated p38. J Exp Med. (2014) 211:1257–70. doi: 10.1084/jem.20131917 PMC4042639 24863062

[191] Gutierrez-Steil C Wrone-Smith T Sun X Krueger JG Coven T Nickoloff BJ. Sunlight-induced basal cell carcinoma tumor cells and ultraviolet-B-irradiated psoriatic plaques express Fas ligand (CD95L). J Clin Invest. (1998) 101:33–9. doi: 10.1172/jci1165 PMC508537 9421463

[192] Hart PH Gorman S Finlay-Jones JJ. Modulation of the immune system by UV radiation: more than just the effects of vitamin D? Nat Rev Immunol. (2011) 11:584–96. doi: 10.1038/nri3045 21852793

[193] Gossec L Kerschbaumer A Ferreira RJO Aletaha D Baraliakos X Bertheussen H. EULAR recommendations for the management of psoriatic arthritis with pharmacological therapies: 2023 update. Ann Rheum Dis. (2024) 83:706–19. doi: 10.1136/ard-2024-225531 PMC11103320 38499325

[194] Du H Liu P Zhu J Lan J Li Y Zhang L. Hyaluronic acid-based dissolving microneedle patch loaded with methotrexate for improved treatment of psoriasis. ACS Appl Mater Interf. (2019) 11:43588–98. doi: 10.1021/acsami.9b15668 31651148

[195] Mercader-García P Silvestre JF Navarro-Triviño FJ Giménez-Arnau AM Pastor-Nieto MA Cordoba-Guijarro S. A re-assessment of the value of markers of corticosteroid contact allergy in the Spanish baseline series: Clobetasol propionate in the spotlight. Contact Dermatitis. (2024) 91:228–36. doi: 10.1111/cod.14639 38965446

[196] Tuckermann JP Kleiman A Moriggl R Spanbroek R Neumann A Illing A. Macrophages and neutrophils are the targets for immune suppression by glucocorticoids in contact allergy. J Clin Invest. (2007) 117:1381–90. doi: 10.1172/jci28034 PMC1849982 17446934

[197] Tatu AL. Topical steroid induced facial rosaceiform dermatitis. Acta Endocrinol (Buchar). (2016) 12:232–3. doi: 10.4183/aeb.2016.232 PMC6535301 31149094

[198] Tokuyama M Mabuchi T. New treatment addressing the pathogenesis of psoriasis. Int J Mol Sci. (2020) 21:7488. doi: 10.3390/ijms21207488 33050592 PMC7589905

[199] Blauvelt A Papp KA Griffiths CE Randazzo B Wasfi Y Shen YK. Efficacy and safety of guselkumab, an anti-interleukin-23 monoclonal antibody, compared with adalimumab for the continuous treatment of patients with moderate to severe psoriasis: Results from the phase III, double-blinded, placebo- and active comparator-controlled VOYAGE 1 trial. J Am Acad Dermatol. (2017) 76:405–17. doi: 10.1016/j.jaad.2016.11.041 28057360

[200] Mease PJ McInnes IB Tam LS Rajalingam R Peterson S Hassan F. Comparative effectiveness of guselkumab in psoriatic arthritis: updates to a systematic literature review and network meta-analysis. Rheumatol (Oxford). (2023) 62:1417–25. doi: 10.1093/rheumatology/keac500 PMC10070072 36102818

[201] Yu C Geng S Yang B Deng Y Li F Kang X. Tildrakizumab for moderate-to-severe plaque psoriasis in Chinese patients: A 12-week randomized placebo-controlled phase III trial with long-term extension. Chin Med J (Engl). (2024) 137:1190–8. doi: 10.1097/cm9.0000000000002873 PMC11101224 38192233

[202] Gordon KB Strober B Lebwohl M Augustin M Blauvelt A Poulin Y. Efficacy and safety of risankizumab in moderate-to-severe plaque psoriasis (UltIMMa-1 and UltIMMa-2): results from two double-blind, randomised, placebo-controlled and ustekinumab-controlled phase 3 trials. Lancet. (2018) 392:650–61. doi: 10.1016/s0140-6736(18)31713-6 30097359

[203] Gooderham M Pinter A Ferris LK Warren RB Zhan T Zeng J. Long-term, durable, absolute Psoriasis Area and Severity Index and health-related quality of life improvements with risankizumab treatment: a *post hoc* integrated analysis of patients with moderate-to-severe plaque psoriasis. J Eur Acad Dermatol Venereol. (2022) 36:855–65. doi: 10.1111/jdv.18010 PMC9314097 35174556

[204] Blauvelt A Kimball AB Augustin M Okubo Y Witte MM Capriles CR. Efficacy and safety of mirikizumab in psoriasis: results from a 52-week, double-blind, placebo-controlled, randomized withdrawal, phase III trial (OASIS-1). Br J Dermatol. (2022) 187:866–77. doi: 10.1111/bjd.21743 PMC10087045 35791755

[205] Papp K Warren RB Green L Reich K Langley RG Paul C. Safety and efficacy of mirikizumab versus secukinumab and placebo in the treatment of moderate-to-severe plaque psoriasis (OASIS-2): a phase 3, multicentre, randomised, double-blind study. Lancet Rheumatol. (2023) 5:e542–52. doi: 10.1016/s2665-9913(23)00120-0 38251498

[206] Langley RG Elewski BE Lebwohl M Reich K Griffiths CE Papp K. Secukinumab in plaque psoriasis–results of two phase 3 trials. N Engl J Med. (2014) 371:326–38. doi: 10.1056/NEJMoa1314258 25007392

[207] Reich K Sullivan J Arenberger P Jazayeri S Mrowietz U Augustin M. Secukinumab shows high and sustained efficacy in nail psoriasis: 2.5-year results from the randomized placebo-controlled TRANSFIGURE study. Br J Dermatol. (2021) 184:425–36. doi: 10.1111/bjd.19262 32479641

[208] Griffiths CE Reich K Lebwohl M van Kerkhof P Paul C Menter A. Comparison of ixekizumab with etanercept or placebo in moderate-to-severe psoriasis (UNCOVER-2 and UNCOVER-3): results from two phase 3 randomised trials. Lancet. (2015) 386:541–51. doi: 10.1016/s0140-6736(15)60125-8 26072109

[209] Kostareva O Svoeglazova A Kolyadenko I Nikulin A Evdokimov S Dzhus U. Two epitope regions revealed in the complex of IL-17A and anti-IL-17A V(H)H domain. Int J Mol Sci. (2022) 23:14904. doi: 10.3390/ijms232314904 36499233 PMC9738047

[210] Lebwohl M Strober B Menter A Gordon K Weglowska J Puig L. Phase 3 studies comparing brodalumab with ustekinumab in psoriasis. N Engl J Med. (2015) 373:1318–28. doi: 10.1056/NEJMoa1503824 26422722

[211] von Kiedrowski R Hinz T Mauer G Schwinn A Timmel A Hutt HJ. Management of moderate to severe psoriasis with brodalumab-Real-world evidence from the LIBERO study. J Eur Acad Dermatol Venereol. (2024) 38:2156–66. doi: 10.1111/jdv.19974 38572773

[212] Leonardi CL Kimball AB Papp KA Yeilding N Guzzo C Wang Y. Efficacy and safety of ustekinumab, a human interleukin-12/23 monoclonal antibody, in patients with psoriasis: 76-week results from a randomised, double-blind, placebo-controlled trial (PHOENIX 1). Lancet. (2008) 371:1665–74. doi: 10.1016/s0140-6736(08)60725-4 18486739

[213] Grine L Dejager L Libert C Vandenbroucke RE. An inflammatory triangle in psoriasis: TNF, type I IFNs and IL-17. Cytokine Growth Factor Rev. (2015) 26:25–33. doi: 10.1016/j.cytogfr.2014.10.009 25434285

[214] Mylonas A Conrad C. Psoriasis: classical vs. Paradoxical. The yin-yang of TNF and type I interferon. Front Immunol. (2018) 9:2746. doi: 10.3389/fimmu.2018.02746 30555460 PMC6283263

[215] Duan Y Sun W Li Y Shi Z Li L Zhang Y Spirohypertones A. and B as potent antipsoriatics: Tumor necrosis factor-α inhibitors with unprecedented chemical architectures. Acta Pharm Sin B. (2024) 14:2646–56. doi: 10.1016/j.apsb.2024.02.002 PMC11143743 38828134

[216] Kolahdooz H Khori V Erfani-Moghadam V Livani F Mohammadi S Memarian A. Niosomal curcumin suppresses IL17/IL23 immunopathogenic axis in skin lesions of psoriatic patients: A pilot randomized controlled trial. Life (Basel). (2023) 13:1076. doi: 10.3390/life13051076 37240721 PMC10224439

[217] Zhang C Shestopaloff K Hollis B Kwok CH Hon C Hartmann N. Response to anti-IL17 therapy in inflammatory disease is not strongly impacted by genetic background. Am J Hum Genet. (2023) 110:1817–24. doi: 10.1016/j.ajhg.2023.08.010 PMC10577077 37659414

[218] Xu B Yu B Xu Z Ye S Qing Y Sun H. Investigation and confirmation of PYCARD as a potential biomarker for the management of psoriasis disease. J Inflammation Res. (2024) 17:6415–37. doi: 10.2147/jir.S468746 PMC11414756 39310902

[219] Villanova F Di Meglio P Nestle FO. Biomarkers in psoriasis and psoriatic arthritis. Ann Rheum Dis. (2013) 72 Suppl 2:ii104–110. doi: 10.1136/annrheumdis-2012-203037 23532439

[220] Garshick MS Ward NL Krueger JG Berger JS. Cardiovascular risk in patients with psoriasis: JACC review topic of the week. J Am Coll Cardiol. (2021) 77:1670–80. doi: 10.1016/j.jacc.2021.02.009 PMC8168628 33795041

[221] FitzGerald O Gladman DD Mease PJ Ritchlin C Smolen JS Gao L. Phase 2 trial of deucravacitinib in psoriatic arthritis: biomarkers associated with disease activity, pharmacodynamics, and clinical responses. Arthritis Rheumatol. (2024) 76:1397–407. doi: 10.1002/art.42921 38770592

[222] Ademowo OS Hernandez B Collins E Rooney C Fearon U van Kuijk AW. Discovery and confirmation of a protein biomarker panel with potential to predict response to biological therapy in psoriatic arthritis. Ann Rheum Dis. (2016) 75:234–41. doi: 10.1136/annrheumdis-2014-205417 25187158

[223] Wagner CL Visvanathan S Elashoff M McInnes IB Mease PJ Krueger GG. Markers of inflammation and bone remodelling associated with improvement in clinical response measures in psoriatic arthritis patients treated with golimumab. Ann Rheum Dis. (2013) 72:83–8. doi: 10.1136/annrheumdis-2012-201697 PMC3551220 22975755

[224] Chan BCL Lam CWK Tam LS Wong CK. IL33: roles in allergic inflammation and therapeutic perspectives. Front Immunol. (2019) 10:364. doi: 10.3389/fimmu.2019.00364 30886621 PMC6409346

[225] Zhou T Zhang S Zhou Y Lai S Chen Y Geng Y. Curcumin alleviates imiquimod-induced psoriasis in progranulin-knockout mice. Eur J Pharmacol. (2021) 909:174431. doi: 10.1016/j.ejphar.2021.174431 34428436

[226] Huh JR Leung MW Huang P Ryan DA Krout MR Malapaka RR. Digoxin and its derivatives suppress TH17 cell differentiation by antagonizing RORγt activity. Nature. (2011) 472:486–90. doi: 10.1038/nature09978 PMC3172133 21441909

[227] Laurindo LF de Carvalho GM de Oliveira Zanuso B Figueira ME Direito R de Alvares Goulart R. Curcumin-based nanomedicines in the treatment of inflammatory and immunomodulated diseases: an evidence-based comprehensive review. Pharmaceutics. (2023) 15:229. doi: 10.3390/pharmaceutics15010229 36678859 PMC9861982

[228] Menter A Korman NJ Elmets CA Feldman SR Gelfand JM Gordon KB. Guidelines of care for the management of psoriasis and psoriatic arthritis: section 6. Guidelines of care for the treatment of psoriasis and psoriatic arthritis: case-based presentations and evidence-based conclusions. J Am Acad Dermatol. (2011) 65:137–74. doi: 10.1016/j.jaad.2010.11.055 21306785

[229] Wu D Hailer AA Wang S Yuan M Chan J El Kurdi A. A single-cell atlas of IL-23 inhibition in cutaneous psoriasis distinguishes clinical response. Sci Immunol. (2024) 9:eadi2848. doi: 10.1126/sciimmunol.adi2848 38277466

[230] Papp KA Blauvelt A Bukhalo M Gooderham M Krueger JG Lacour JP. Risankizumab versus ustekinumab for moderate-to-severe plaque psoriasis. N Engl J Med. (2017) 376:1551–60. doi: 10.1056/NEJMoa1607017 28423301

[231] Meglio P Di Cesare A Laggner U Chu CC Napolitano L Villanova F. The IL23R R381Q gene variant protects against immune-mediated diseases by impairing IL-23-induced Th17 effector response in humans. PloS One. (2011) 6:e17160. doi: 10.1371/journal.pone.0017160 21364948 PMC3043090

[232] Ramón-Vázquez A Flood P Cashman TL Patil P Ghosh S. T lymphocyte plasticity in chronic inflammatory diseases: The emerging role of the Ikaros family as a key Th17-Treg switch. Autoimmun Rev. (2025) 24:103735. doi: 10.1016/j.autrev.2024.103735 39719186

[233] Olejniczak-Staruch I Ciążyńska M Sobolewska-Sztychny D Narbutt J Skibińska M Lesiak A. Alterations of the skin and gut microbiome in psoriasis and psoriatic arthritis. Int J Mol Sci. (2021) 22:3998. doi: 10.3390/ijms22083998 33924414 PMC8069836

[234] Buhaş MC Gavrilaș LI Candrea R Cătinean A Mocan A Miere D. Gut microbiota in psoriasis. Nutrients. (2022) 14:2970. doi: 10.3390/nu14142970 35889927 PMC9321451

[235] Ferrara F Verduci C Laconi E Mangione A Dondi C Del Vecchio M. Current therapeutic overview and future perspectives regarding the treatment of psoriasis. Int Immunopharmacol. (2024) 143:113388. doi: 10.1016/j.intimp.2024.113388 39405929

